# Short-Term Effects of Kefir-Fermented Milk Consumption on Bone Mineral Density and Bone Metabolism in a Randomized Clinical Trial of Osteoporotic Patients

**DOI:** 10.1371/journal.pone.0144231

**Published:** 2015-12-10

**Authors:** Min-Yu Tu, Hsiao-Ling Chen, Yu-Tang Tung, Chao-Chih Kao, Fu-Chang Hu, Chuan-Mu Chen

**Affiliations:** 1 Department of Life Sciences, and Agricultural Biotechnology Center, National Chung Hsing University, Taichung 402, Taiwan; 2 Department of Orthopaedic Surgery, Taichung Armed Forces General Hospital, Taichung 411, Taiwan; 3 Department of Biomedical Engineering, Hungkuang University, Taichung 433, Taiwan; 4 School of Medicine, National Defense Medical Center, Taipei 114, Taiwan; 5 Department of Bioresources, Da-Yeh University, Changhua 515, Taiwan; 6 Division of Biostatistics, Graduate Institute of Clinical Medicine and School of Nursing College of Medicine, National Taiwan University, Taipei 100, Taiwan; 7 Rong Hsing Research Center for Translational Medicine, and the iEGG Center, National Chung Hsing University, Taichung 402, Taiwan; University of Ottawa, CANADA

## Abstract

Milk products are good sources of calcium that may reduce bone resorption and help prevent bone loss as well as promote bone remodeling and increase bone formation. Kefir is a product made by kefir grains that degrade milk proteins into various peptides with health-promoting effects, including antithrombotic, antimicrobial and calcium-absorption enhancing bioactivities. In a controlled, parallel, double-blind intervention study over 6 months, we investigated the effects of kefir-fermented milk (1,600 mg) supplemented with calcium bicarbonate (CaCO_3_, 1,500 mg) and bone metabolism in 40 osteoporosis patients, and compared them with CaCO_3_ alone without kefir supplements. Bone turnover markers were measured in fasting blood samples collected before therapy and at 1, 3, and 6 months. Bone mineral density (BMD) values at the spine, total hip, and hip femoral neck were assessed by dual-energy x-ray absorptiometry (DXA) at baseline and at 6 months. Among patients treated with kefir-fermented milk, the relationships between baseline turnover and 6 months changes in DXA-determined BMD were significantly improved. The serum β C-terminal telopeptide of type I collagen (β-CTX) in those with T-scores > -1 patients significantly decreased after three months treatment. The formation marker serum osteocalcin (OC) turned from negative to positive after 6 months, representing the effect of kefir treatment. Serum parathyroid hormone (PTH) increased significantly after treatment with kefir, but decreased significantly in the control group. PTH may promote bone remodeling after treatment with kefir for 6 months. In this pilot study, we concluded that kefir-fermented milk therapy was associated with short-term changes in turnover and greater 6-month increases in hip BMD among osteoporotic patients.

**Trial Registration:** ClinicalTrials.gov NCT02361372

## Introduction

Osteoporosis is a chronic disease, also called a “silent disease” or “quiet epidemic” that occurs in elderly people and leads to easy bone fracture [1–5]. Osteoporotic fractures are a major cause of morbidity, causing more months of disability than cancer [6], and are associated with increased mortality. They also contribute substantially to health care costs worldwide [7]. Effective methods to reduce fracture risk are therefore likely to benefit population health. Osteoporosis prevention is of eminent interest because of the increasing global relevance of this bone disease.

Inadequate intake of calcium accelerates loss of bone mass, raising the risk for osteoporotic fractures, especially in women after menopause [8,9]. Due to the reduced intestinal calcium absorption capacity and renal function with increasing age and to the accelerated loss of bone mass in females because of postmenopausal estrogen deficiency, when bone turnover increases, dietary calcium requirement also increases [10]. Nutritional supplements such as vitamins [11], protein [12,13], and amino acids [14] are now known to promote bone remodeling and to enhance calcium absorption, consequently inhibiting osteoporosis.

Heaney [15] summarized in his review that nearly all controlled intervention studies and approximately 75% of observational studies indicated an improvement in bone health with dietary calcium. Milk products are rich in calcium and have high bioavailability [15,16]. Thus, milk products are considered optimal for building up bone tissue during growth and attenuating loss of bone mineral throughout life. For casein phosphopeptides (CPPs), deriving from tryptic hydrolysis of casein, results are conflicting; for review see [17,18]. In rats, some studies found that CPPs enhanced calcium absorption and/or improved calcium incorporation into bone [18,19]. CPPs are a large group of peptides with a phosphoseryl residue in common. Phosphopeptides are formed either from casein by proteolytic enzymes during fermentation or in the gastrointestinal tract. CPPs increase calcium absorption by forming a hydrophobic complex with calcium, thus preventing the formation of insoluble calcium phosphates [20]. *In vitro* studies have shown that CPPs affect calcium absorption by inhibiting the precipitation of calcium in the intestine [19]. On the other hand, the amounts of Ca absorbed increased from the CPP/Ca ratio of 5 to a ratio of 15 and decreased with a ratio of 20. The excess CPPs might have formed larger complexes that hid the Ca ion and, hence, impaired mineral release and decreased its availability [21]. Similar reductions of Ca absorption have been found in rats fed one meal with CPP/Ca ratios of 40 to 100 compared with one protein-free meal [22].

Originating from the North Caucasus, kefir-fermented milk has been confirmed in multiple studies to include probiotics formed by bacteria and yeast. The health functions of kefir have been mostly verified, including improved intestinal flora and lactose intolerance; enhanced immune function, inhibitory activity, antitumor activity and antioxidant effects; and reduced cholesterol. Studies have found that milk fermented with *Lactobacillus helveticus* can increase calcium metabolism or slow calcium loss in postmenopausal women [23]. Another study confirmed that fermented milk can reduce bone resorption at night in postmenopausal women [24]. Elucidating the effects of osteoporosis and nutrition supplements with kefir-fermented milk on bone mineral density (BMD) is important in clinical practice. Therefore, clarify the correlations between BMD and bone turnover markers and treatment with kefir-fermented milk to prevent or delay osteoporosis need to be validate in clinical trial.

In this controlled, parallel, double-blind intervention study, we investigated the effects on bone metabolism of kefir-fermented milk supplemented with calcium carbonate (CaCO_3_) versus placebo with CaCO_3_. The hypothesis was that consumption of kefir-fermented milk inhibits bone resorption and promotes optimal bone formation.

## Materials and Methods

### Kefir preparation

Kefir starter grains were inoculated (5%, wt/vol) and propagated in sterilized milk at 20°C for 20 h to activate them. The grains were retrieved through a sieve, reinoculated (10%, wt/vol) into sterilized fresh milk, and the incubation conditions were performed according to our previous reports [25,26]. After the grains were filtered, the fermented products were freeze-dried as a kefir powder using a freeze dryer (VirTis, Warminster, PA, USA). The content of peptides in kefir calculated as triglycine equivalent in milligrams per gram sample was 21.39 mg/g.

### Study participants

The clinical trial design was approved by the Institutional Review Board of Tri-Service General Hospital Medical Center (TSGH-IRB TC098-13; as shown in S1 Appendix) and ClinicalTrials.gov (NCT02361372; as shown in S2 Appendix). The Taichung Armed Forces General Hospital approved this study on May 19, 2010. The recruitment period was from May, 2010 to July, 2011. Participant recruitments were interested in the study, and asked to read through the informed consent form. Participants were followed for six months, and the follow-up survey was held on November, 2010 to January, 2012. The medical records of 69 osteoporosis patients were screened for inclusion and exclusion criteria. The inclusion criteria were (1) a BMD dual-energy X-ray absorptiometer (DXA) T-score of -2.5 or less at the femoral neck, with or without evidence of existing vertebral fracture, or a T-score of -1.5 or less, with radiologic evidence of at least two mild vertebral fractures or one moderate vertebral fracture; and (2) BMD T-score of < -2.5 or diagnosis with fractures in the spine, hip, wrist or other positions caused by osteoporosis through X-ray examination. Exclusion criteria in Taichung Armed Forces General Hospital approval study as follow: (1) those who had bilateral ovariectomies and who had natural menopause before 40 years old; (2) those who use drugs or drugs for bone metabolism, such as glucocorticoid, thyroxin, antiepileptics, bisphosphonates, calcitonin, strontium ranelate, and hormone replacement therapy for more than 4 months, such as estrogen, parathyroid hormone 1–34, teriparatide (PTH); (3) those whose weight is over 100 kilograms; (4) those who suffered from the following primary or secondary diseases: hyperparathyroidism, hyperthyroidism, diabetes, cirrhosis, and kidney failure, all of whom have medical history or are established by laboratory reports (S3 Appendix).

**Fig 1:**
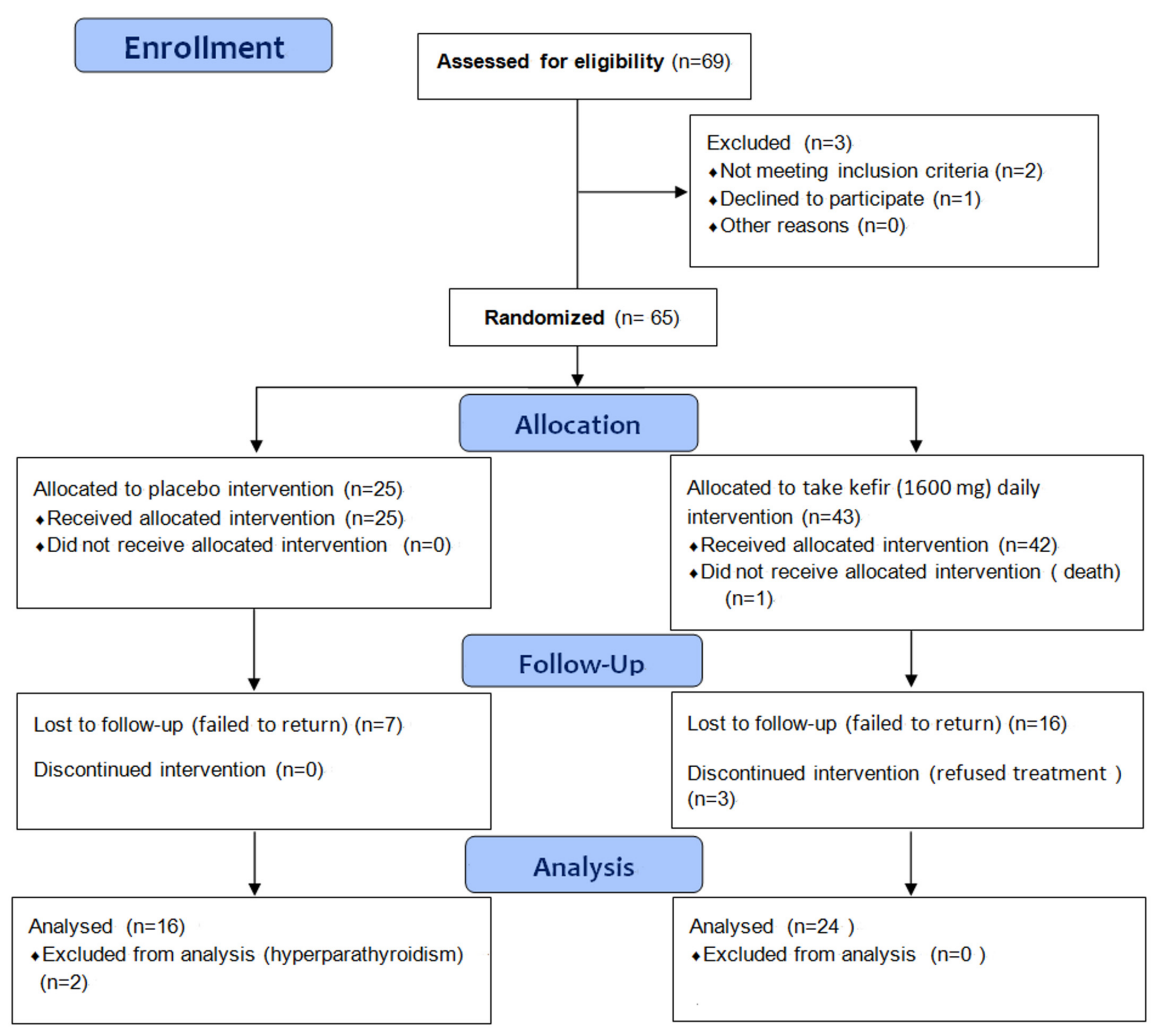
Flow diagram of patient recruitment and follow-up.

All patients gave written informed consent to participate. Fig 1 showed the flow diagram of patient recruitment and follow-up. 40 patients with osteoporosis were enrolled in this study. Of the 40 osteoporosis patients, 16 in the control group and 24 in the treatment group completed the 6-month study. The patients were aged 67.94 ± 8.37 years in the control group and 64.08 ± 14.51 years in the treatment group. Those in the control group had a height of 157.48 ± 8.68 cm and those in the treatment group were 154.88 ± 7.92 cm. Subjects weighed an average of 60.50 ± 11.85 kg in the control group and 57.51 ± 8.51 kg in the treatment group (Table 1).

**Table 1:** Effects of kefir consumption on clinical assessment in osteoporotic patients.^1^

	Control	Treatment	
Variable	(*n* = 16)	(*n* = 24)	*p* value
Male, *n* (%)	7 (43.8%)	7 (29.2%)	0.500^†^
Age (years)	67.94 ± 8.37	64.08 ± 14.51	0.263^§^
Height (cm)	157.48 ± 8.68	154.88 ± 7.92	0.639
Weight (kg)	60.50 ± 11.85	57.51 ± 8.51	0.553
Dual-energy x-ray absorptiometry T-score groups			0.232
T-score ≤ −2.5	2 (18.2%)	9 (81.8%)	
−2.5 < T-score ≤ −1	11 (47.8%)	12 (52.2%)	
−1 < T-score	3 (50.0%)	3 (50.0%)	
β C-terminal telopeptide of type I collagen (pg/ml)			
Baseline level	420.10 ± 248.96	456.32 ± 334.66	0.989
1 month after treatment	373.55 ± 220.58	397.55 ± 231.78	0.859
3 months after treatment	328.40 ± 194.88	369.55 ± 225.19	0.633
6 months after treatment	330.72 ± 153.72	425.22 ± 206.88	0.180
Osteocalcin (ng/ml)			
Baseline level	19.14 ± 7.87	19.65 ± 10.24	0.946
1 month after treatment	17.80 ± 6.87	16.65 ± 7.23	0.521
3 months after treatment	15.96 ± 6.14	16.67 ± 8.95	0.817
6 months after treatment	14.44 ± 5.18	16.86 ± 6.19	0.325
Spine BMD (gm/cm^2^)			
Baseline level	0.842 ± 0.215	0.843 ± 0.193	0.872
6 months after treatment	0.852 ± 0.204	0.849 ± 0.201	0.909
Spine T-score			
Baseline level	-1.233 ± 1.954	-1.294 ± 1.698	0.976
6 months after treatment	-0.833 ± 1.615	-1.202 ± 1.752	0.740
Femoral neck BMD (gm/cm^2^)			
Baseline level	0.629 ± 0.143	0.560 ± 0.139	0.439
6 months after treatment	0.635 ± 0.126	0.591 ± 0.148	0.501
Femoral neck T-score			
Baseline level	-1.657 ± 1.305	-2.328 ± 1.252	0.431
6 months after treatment	-1.669 ± 1.181	-2.003 ± 1.343	0.791
Total hip BMD (gm/cm^2^)			
Baseline level	0.742 ± 0.161	0.677 ± 0.173	0.596
6 months after treatment	0.753 ± 0.153	0.689 ± 0.182	0.520
Total hip T score			
Baseline level	-0.973 ± 1.303	-1.645 ± 1.419	0.395
6 months after treatment	-0.871 ± 1.183	-1.534 ± 1.498	0.417
GOT (U/L)			
Baseline level	22.688 ± 6.183	24.292 ± 11.615	0.781
1 month after treatment	22.500 ± 5.854	25.167 ± 12.792	0.739
6 months after treatment	21.429 ± 5.840	25.261 ± 14.961	0.694
GPT (U/L)			
Baseline level	20.323 ± 8.039	23.792 ± 21.925	0.792
1 month after treatment	18.813 ± 6.014	23.208 ± 14.850	0.628
6 months after treatment	18.214 ± 8.377	19.870 ± 14.639	0.801
Alkaline phosphatase (IU/L)			
Baseline level	76.000 ± 21.541	73.333 ± 23.766	0.619
1 month after treatment	71.250 ± 20.434	72.500 ± 25.260	0.868
6 months after treatment	64.786 ± 18.289	70.261 ± 21.672	0.471
Albumin (g/dL)			
Baseline level	4.531 ± 0.250	4.446 ± 0.527	0.824
1 month after e treatment	4.525 ± 0.221	4.492 ± 0.306	0.978
6 months after treatment	4.429 ± 0.425	4.509 ± 0.325	0.591
Creatinine (mg/dL)			
Baseline level	0.776 ± 0.186	0.758 ± 0.265	0.481
1 month after treatment	0.790 ± 0.207	0.766 ± 0.302	0.369
6 months after treatment	0.753 ± 0.204	0.802 ± 0.375	0.851
Homocysteine (μmol/L)			
Baseline level	12.213 ± 3.366	10.565 ± 3.312	0.134
1 month after treatment	11.928 ± 2.927	11.039 ± 3.051	0.258
6 months after treatment	13.219 ± 3.741	11.402 ± 3.344	0.169
Vitamin D 25-OH (ng/ml)			
Baseline level	21.203 ± 11.735	25.245 ± 13.039	0.292
6 months after treatment	26.754 ± 15.495	18.700 ± 9.852	0.147
Calcium (mg/dL)			
Baseline level	9.019 ± 0.302	9.104 ± 0.320	0.248
1 month after treatment	9.156 ± 0.331	9.100 ± 0.299	0.485
6 months after e treatment	8.885 ± 0.864	9.204 ± 0.299	0.251
Phosphorus (mg/dL)			
Baseline level	3.825 ± 0.478	3.938 ± 0.350	0.608
1 month after treatment	3.638 ± 0.318	3.733 ± 0.516	0.560
6 months after treatment	3.636 ± 0.488	3.735 ± 0.440	0.604
Cortisol (μg/dL)			
Baseline level	9.511 ± 3.952	9.485 ± 3.630	0.571
1 month after treatment	9.428 ± 3.768	8.033 ± 4.224	0.233
6 months after treatment	9.109 ± 4.565	8.583 ± 2.857	0.684
Testosterone (ng/ml)			
Baseline level	1.187 ± 2.610	1.104 ± 2.390	0.315
1 month after treatment	1.599 ± 2.335	1.054 ± 2.279	0.364
6 months after treatment	1.834 ± 2.854	1.167 ± 2.481	0.382
Estradiol (ng/ml)			
Baseline level	6.371 ± 7.224	8.874 ± 15.450	0.970
1 month after treatment	6.001 ± 6.892	9.629 ± 12.909	0.642
6 months after treatment	5.428 ± 7.280	7.073 ± 7.492	0.318
Parathyroid hormone (pg/ml)			
Baseline level	37.889 ± 13.233	35.025 ± 15.421	0.404
1 month after treatment	39.424 ± 11.927	36.378 ± 13.341	0.345
6 months after treatment	34.014 ± 15.079	41.901 ± 14.054	0.107

^1^The sample statistics presented in this table were mean ± standard deviation (SD) for continuous variables and frequency (percentage, %) for categorical variables. The listed *p*-values of statistical tests were calculated using the Wilcoxon rank-sum test for continuous variables and the Fisher’s exact test for categorical variables.

^†^ Fisher’s exact test *p* value for categorical variables.

^§^ Wilcoxon rank sums test *p* value for continuous variables.

### Nutrition supplements and drug delivery

In this controlled, parallel, double-blind intervention study, patients diagnosed with osteoporosis were divided into two groups. Patients in the kefir-fermented milk treatment group were oral administered 1,600 mg kefir-fermented milk [25,26] per day and an accompanying supplement of 1,500 mg CaCO_3_. Control group patients were supplemented with placebo (1,600 mg unfermented raw milk) and 1,500 mg of CaCO_3_ daily. The internal calcium content of kefir-fermented milk and raw milk are estimated as 56.6 mg/g [26]. Compared to the supplement of 1,500 mg CaCO_3_, the additional calcium intake/dietary calcium is 5.36%. Thus, the additional calcium could be ignored. According to the allocation table, a label indicating the corresponding number was attached to each plastic of treatments. After treatments were allocated, the Registration Center sealed and kept the allocation table in confidence until the clinical trial was completed. Investigators at each facility checked all inclusion and exclusion criteria and registered patients one at a time by faxing patient information obtained under informed consent to the Registration Center. The center again checked the documents to make sure that each subject had satisfied all inclusion and exclusion criteria, then randomly allocated subjects as necessary to receive treatments based on a single block consisting of one sample each from different groups. The assigned treatment numbers were then faxed back to the investigators. The blind was not broken until this clinical trial was completely finished (S4 Appendix).

### Clinical assessment

Venous blood was taken from patients (10 ml each) at baseline and during the first, third and sixth months of treatments. Biochemical tests were made to determine the levels of substances affecting calcium metabolism, including concentrations of calcium and phosphorus, parathyroid hormone (PTH), glutamate oxaloacetate transaminase (GOT), glutamate pyruvate transaminase (GPT), alkaline phosphatase (ALP), albumin, creatinine, homocysteine, vitamin D 25-OH, cortisol, testosterone, and estradiol [27,28]. Safety was assessed by the recording of all adverse events and by physical examination, regular measurement of vital signs, and regular monitoring of hematologic and blood chemical values when blood was taken.

The markers of bone turnover, β C-terminal telopeptide of type I collagen (β-CTX) and osteocalcin (OC), were measured at baseline, 1, 3, and 6 months. Blood was collected after an overnight fast, and serum stored at −70°C until analyzed. Measurements were performed at an internationally accredited local laboratory using the Roche Elecsys 2010 platform (Roche Diagnostics, Indianapolis, IN). The coefficient of variation (CVs) of these markers is β-CTX, 5.1%. The serum concentration of OC (OCN-Mid) was measured by a recently developed ELISA (two-site N-MID Osteocalcin A/S, Denmark) [17]. The intra- and inter-assay coefficients of variation were less than 4.2% and 4.0%, respectively, and the detection limit was 2.0 ng/ml. BMD was measured at baseline and at 6 months at the lumbar spine (L1-L4), total hip, and femoral neck, using a DXA (QDR 4500W DOS series bone densitometer, Hologic, Bedford, MA).

### Statistical analysis

Statistical analyses were performed with SAS software, Version 9.1.3 (SAS Institute Inc., Cary, NC). Two-sided *P* ≤ 0.05 was considered statistically significant. Group differences were assessed by Chi-square test or Fisher’s exact test for categorical variables and by two-sample t-test or Wilcoxon rank-sum test for continuous variables.

We performed the intent-to-treat (ITT) analysis by testing the difference in the mean values of the end points between the treatment and placebo groups of patients, and then listed the testing results in Table 1. In addition to the ITT analysis, we also performed per protocol (PP) analysis by conducting regression analysis on each of the end point. Regression analysis helped us control any unexpected baseline imbalances in the distributions of confounding variables and gain the efficiency in estimating the treatment effect due to the reduction of the error variance of the fitted regression model.

To investigate the predictive values of age, sex, Ca, β-CTX, and OC on changes from baseline in spine BMD, femoral neck BMD, and total hip BMD, we conducted regression analyses. First, we carried out the logistic regression analysis with only the first episode of each patient. Then, we fitted a marginal logistic regression model using the generalized estimating equations (GEE) method for all observed episodes of each patient. The GEE method helped us obtain consistent estimates of the standard errors for the estimated regression coefficients from the correlated data so that we could make consistent inferences on the regression coefficients based on the correct *p* values. In the GEE analysis, if the first-order autocorrelation (AR [1]) structure fitted the repeated measures data well, the model-based estimates of the standard errors for the estimated regression coefficients were used; otherwise, the empirical standard error estimates were reported, as long as the sample size was large.

The goal of regression analysis was to find one or a few *parsimonious* regression models that fit the observed data well. To ensure the quality of analysis results, basic model-fitting techniques for (1) variable selection, (2) goodness-of-fit (GOF) assessment, and (3) regression diagnostics were used. Specifically, the stepwise variable selection procedure was applied to obtain the candidate final regression model. All the univariate significant and non-significant relevant covariates were selected and the significance levels for entry (SLE) and for stay (SLS) were set to 0.15 or larger. Then, with the aid of substantive knowledge, the best final regression model was identified manually by reducing the significance levels to 0.05. Any discrepancy between the results of univariate analysis and multivariate analysis was likely due to the confounding effects of the uncontrolled covariates in the univariate analysis. Both the GOF measures (including the percentage of concordant pairs, estimated area under the receiver operating characteristic (ROC) curve, and adjusted generalized *R* ^2^) and the GOF tests (including the deviance GOF test, Pearson chi-squared GOF test, and Hosmer-Lemeshow GOF test) were examined to assess the GOF of the fitted logistic regression model. Even so, the value of the adjusted generalized *R* ^2^ for the logistic regression model was usually low. Larger *p* values for the deviance GOF test, Pearson chi-squared GOF test, and Hosmer-Lemeshow GOF test indicated better fits. We also used the regression diagnostic tools such as residual analysis, detection of influential cases, and check for multicollinearity to uncover any model or data problems.

## Results

### Patient characteristics and adherence

Of the 69 patients recruited into the trial, 40 completed the study. The baseline characteristics of the participants were entered the original protocol (Table 1). No significant difference was found between the two groups in terms of average age, sex, height, weight, bone turnover markers (β-CTX and OC), or BMD. The average BMD increased in both two groups after treatment, especially in the kefir group (in which the hip femoral neck bone density increased from 0.560 ± 0.139 gm/cm^2^ to 0.591 ± 0.148 gm/cm^2^), but the median was not statistically different between the two groups. Bone turnover markers (β-CTX and OC) decreased compared to baseline values, but the median was not statistically different between the two groups. Biochemical tests of elements affecting calcium metabolism (calcium and phosphorus, PTH, GOT, GPT, ALP, albumin, creatinine, homocysteine, Vitamin D 25-OH, cortisol, testosterone, and estradiol) did not differ significantly between the two groups (Table 1).

### Change in patient’s serum calcium and PTH over time

**Fig 2:**
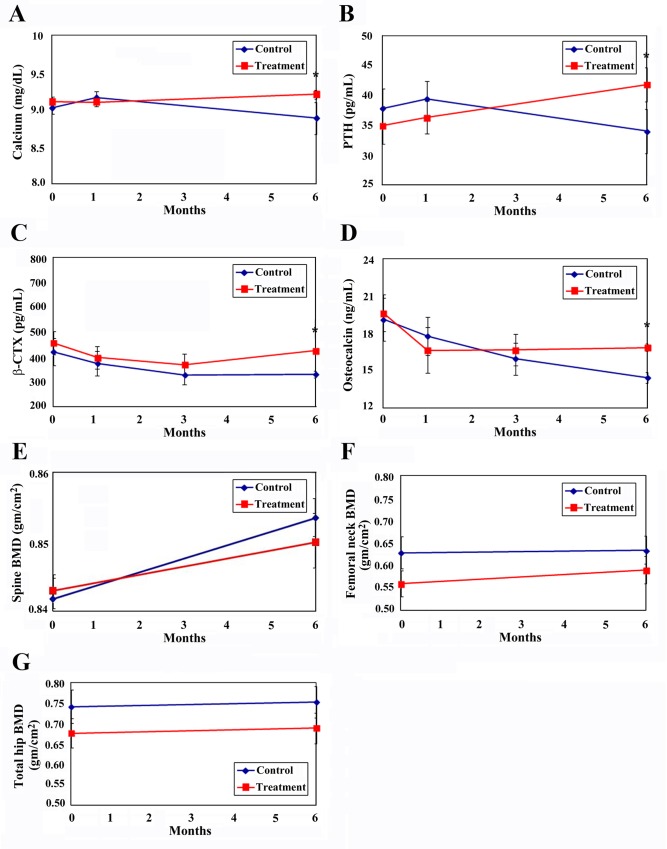
Effects of kefir consumption on clinical assessment in osteoporotic patients. (**A**) Serum calcium. (**B**) Parathyroid hormone (PTH). (**C**) β C-Terminal telopeptide of type I collagen (β-CTX). (**D**) Osteocalcin. (**E**) Spine bone mineral density (BMD). (**F**) Femoral neck BMD. (**G**) Total hip BMD. *P < 0.05 vs. control group.

Serum calcium was slightly increased in patients who received kefir treatment for six months (*P* = 0.251). In the group without kefir treatment, serum calcium was significantly decreased at the sixth month (Fig 2A). Change in serum PTH after six months of kefir treatment is shown in Fig 2B. Serum PTH levels increased in both groups after the first month. After six months, serum PTH was higher in the experiment group than in the control group (*P* < 0.05) (Fig 2B). The increasing serum calcium and PTH in kefir treatment indicates that the consumption of kefir helps maintain higher serum calcium concentrations. Linear regression analysis of control PTH (before treatment) and other variables with a significant impact on PTH levels indicated that taking kefir for six months resulted in a higher average level of PTH (7.532 pg/ml) (Table 2).

**Table 2:** Effects of kefir consumption on multivariate analyses of the predictors of serum calcium and intact parathyroid hormone (PTH) using multiple linear regression models with generalized estimating equations (GEE).^1^

		Robust	Wald’s	
		standard	chi-square	
Covariate	Estimate	error	test	*p* value
**Y1 = Calcium** ^2^				
Intercept	4.166	2.644	2.480	0.115
Serum calcium baseline concentration	0.543	0.287	3.570	0.059
-2.5 < DXA T-score < -1 × 1 month kefir treatment	-0.325	0.089	13.470	**<0.001**
**Y2 = PTH** ^3^				
Intercept	-59.900	32.095	3.480	0.062
PTH baseline concentration	0.500	0.151	10.930	**0.001**
Male	-15.188	3.257	21.740	**<0.001**
Height (cm)	0.524	0.210	6.210	**0.013**
Kefir treatment for 6 months	7.532	2.895	6.770	**0.009**
-1 < DXA T-score × after 6 months treatment	-6.254	2.297	7.410	**0.006**
-2.5 < DXA T-score < -1	5.643	2.972	3.600	**<0.001**

^1^The repeated measures data were analyzed by multiple marginal regression models using the generalized estimating equations (GEE) method to assess the effects of treatment, learning session and trial on escape latency.

^2^Number of observations = 29, the coefficient of determination (*R* ^2^) was 0.145, indicating that the correlation between the observed response value and the predicted value of the response variable was about 0.38.

^3^Number of observations = 33, the coefficient of determination (*R* ^2^) was 0.437, indicating that the correlation between the observed response value and the predicted value of the response variable was about 0.66.

DXA: dual-energy x-ray absorptiometry.

### Change in mean patient serum levels of β-CTX over time

Serum β-CTX declined over time compared to the baseline value in both groups after six months of treatment. Fig 2 shows a decline after treatment, in the first month after treatment, and in the third month after treatment in both the experimental and control groups. However, β-CTX levels increased after the sixth month of kefir treatment. At all four time points, β-CTX levels were significantly higher in the experiment group (*P* < 0.05) than in the control group (Fig 2C). Linear regression analysis using control β-CTX (baseline value) and other variables with a significant impact on β-CTX found that the subjects who received kefir treatment and who had a T-score >-1 had significantly decreased β-CTX after three months of treatment (*P* = 0.01) (Table 3). That is, the ability of kefir treatment to inhibit bone loss was significantly greater for patients with higher bone density who received three months of treatment.

**Table 3:** Effects of kefir consumption on multivariate analyses of the predictors of the concentrations of osteocalcin, estradiol, parathyroid hormone (PTH), and β C-terminal telopeptide of type I collagen (β-CTX) using multiple linear regression models with the generalized estimating equations (GEE) method.^1^

Covariate	Estimate	Robust standard error	Wald’s chi-square test	*p* value
**Y3 = Osteocalcin changes** ^2^				
Intercept	17.2721	7.9849	4.68	**0.0305**
Osteocalcin baseline concentration	-0.4127	0.0441	87.78	**< 0.0001**
-2.5 < T-score Group < -1 × kefir treatment	-2.8168	0.7857	12.85	**0.0003**
T-score Group < -2.5 × 3 month treatment	4.5968	1.2542	13.43	**0.0003**
Estradiol ≥ 14	1.9387	0.8943	4.70	**0.0302**
After 1 month treatment	2.5080	0.7761	10.44	**0.0012**
T-score Group > -1 × 6 months treatment	2.4825	0.9066	7.50	**0.0062**
-2.5 < T-score Group < -1 × 6 months kefir treatment	2.4943	1.3995	3.18	0.0747
T-score Group > -1 × 1 month treatment	-2.0711	0.9668	4.59	**0.0322**
Height (cm)	-0.0928	0.0507	3.35	0.0670
Parathyroid hormone concentration	0.0376	0.0223	2.84	0.0920
**Y4 = β-CTX changes** ^3^				
Intercept	266.5006	102.0886	6.82	**0.0090**
β-CTX baseline concentration	-0.5312	0.0722	54.07	**< 0.0001**
PTH concentration	2.6163	0.7652	11.69	**0.0006**
Weight (kg)	-3.0928	1.5020	4.24	**0.0390**
T-score Group < -2.5 × 1 month treatment	-103.1012	35.9542	8.22	**0.0041**
-2.5 < T-score Group < -1 × 3 month treatment	-60.0015	16.6072	13.05	**0.0003**
T-score Group > -1 × 3 months kefir treatment	-63.7193	24.7921	6.61	**0.0100**

^1^The repeated measures data were analyzed by multiple marginal regression models using the generalized estimating equations (GEE) method to assess the effects of treatment, learning session and trial on escape latency.

^2^Number of observations = 38, the coefficient of determination (*R* ^2^) was 0.579, indicating that the correlation between the observed response value and the predicted value of the response variable was about 0.76.

^3^Number of observations = 39, the coefficient of determination (*R* ^2^) was 0.552, indicating that the correlation between the observed response value and the predicted value of the response variable was about 0.74.

### Change in mean patient serum levels of OC over time

The average serum levels of the bone formation marker OC declined over time during the 6 months. Analysis of the Group Mean Plot of OC is shown in Fig 2D. OC decreased significantly in the experimental group compared to the control group at one month, then gradually increased to levels higher than in the control group at three and six months (*P* < 0.05). OC decreased in the control group from the beginning of the experiment to six months after treatment (Fig 2D). Linear regression analysis using control OC (baseline value) and other variables with a significant impact on OC showed that subjects who received kefir treatment and had T-scores between -1 and -2.5 had significantly decreased OC levels after one month, a value decreases over other groups (coefficient -2.8168, *P* = 0.0003). After treatment for six months, the value turned from negative to positive (coefficient 2.4943, *P* = 0.0747) (Table 3).

The relative timing of these changes in bone markers is consistent with the hypothesis that the initial anabolic activity of kefir affects the processes associated with bone formation. Changes in β-CTX, on the other hand, are delayed for several months, reflecting the subsequent effect on bone remodeling of osteoclast activation. The bone turnover markers β-CTX and OC decreased after treatment. Especially in patients with higher bone density (DXA -1> T-score > -2.5) who received kefir treatment, the bone metabolic indicators were effectively inhibited after one month of treatment. In these groups, however, bone formation indicators (N_MID_Osteocalcin) rose earlier than in those who received calcium only. For kefir-supplemented subjects, blood calcium concentrations were enhanced and serum levels remained high. PTH concentrations were also maintained.

### Change in BMD over time

After kefir treatment for six months, the BMD of the spine and hip as measured by DXA tended to increase. The lumbar spine (L1-L4) BMD increased from 0.843 ± 0.193 g/cm^2^ to 0.849 ± 0.201 g/cm^2^, femoral neck BMD increased significantly by 5.5%, from 0.560 ± 0.139 g/cm^2^ to 0.591 ± 0.1478 g/cm^2^, and total hip BMD increased from 0.677 ± 0.173 g/cm^2^ to 0.689 ± 0.182 g/cm^2^ (Table 1). Fig 2G shows the change in total hip BMD of the experimental group and the control group. Total hip BMD rose in both groups, but was lower in the experimental group than in the control group (Fig 2G). As shown in Fig 2F, the average hip femoral neck BMD was lower in the experimental group than in the control group both before and after treatment for six months, although it rose in the experimental group. Analysis using a linear regression model found that kefir had significant interactions with OC baseline levels (coefficient 0.001; *P* = 0.028). This result means that, on average, the impact of treatment on hip femoral neck BMD was associated with the baseline OC level (Table 4). Fig 2E shows that the average spine BMD of the experimental group was higher than that in the control group at baseline and lower than that in the control group after six months; it increased slightly in both the experimental and the control groups (Fig 2E).

**Table 4:** Effect of kefir consumption on multivariate analyses of the predictors of differences in spine, hip, femoral neck, and total hip bone mineral density (BMD) over time using multiple linear regression models.^1^

Covariate	Estimate	Standard error	*t* test	*p* value
**Y5 = Spine BMD changes** ^2^				
Intercept	0.1820	0.0606	3.003	**0.0063**
6^th^ month intact parathyroid hormone	-0.0008	0.0002	-3.546	**0.0017**
T-score Group > -1	-0.0190	0.0076	-2.497	**0.0203**
Height (cm)	-0.0009	0.0004	-2.241	**0.0350**
3^rd^ month β-CTX concentration × kefir treatment	-3.46×10^−05^	1.96×10^−05^	-1.765	0.0905
**Y6 = Femoral neck BMD changes** ^3^				
Intercept	-0.0319	0.0161	-1.975	**0.0604**
1^st^ month estradiol ≤ 13	0.0500	0.0177	2.831	**0.0095**
Osteocalcin baseline concentration × kefir treatment	0.0013	0.0006	2.338	**0.0284**
**Y7 = Total hip BMD changes** ^**4**^				
Intercept	0.1091	0.0251	4.349	**0.0009**
Total hip BMD before treatment	-0.0625	0.0279	-2.242	**0.0447**
T-score Group > -1	-0.0346	0.0140	-2.466	**0.0297**
T-score Group < -2.5	0.0280	0.0117	2.396	**0.0338**
1^st^ month E2 estradiol ≤ 13	0.0221	0.0077	2.867	**0.0142**
Osteocalcin baseline concentration	-0.0050	0.0008	-6.313	**< 0.0001**
β-CTX baseline concentration	0.0002	4.01×10^−05^	3.828	**0.0024**
1^st^ month β-CTX concentration	-0.0001	3.91×10^−05^	-2.254	**0.0437**
1^st^ month testosterone concentration	-0.0042	0.0016	-2.563	**0.0249**
Kefir treatment	-0.1346	0.0458	-2.941	**0.0123**
Total hip BMD baseline × kefir treatment	0.1531	0.0454	3.374	**0.0055**
β-CTX baseline concentration × kefir treatment	-0.0001	0.0001	-2.309	**0.0396**
1^st^ month β-CTX concentration × kefir treatment	0.0002	0.0001	3.594	**0.0037**
1^st^ month Osteocalcin concentration × kefir treatment	-0.0031	0.0013	-2.452	**0.0305**
Osteocalcin baseline concentration × kefir treatment	0.0031	0.0013	2.386	**0.0344**

^1^The repeated measures data were analyzed by multiple marginal regression models using the generalized estimating equations (GEE) method to assess the effects of treatment, learning session and trial on escape latency.

^2^Number of observations = 28, the coefficient of determination (*R* ^2^) was 0.516, indicating that the correlation between the observed response value and the predicted value of the response variable was about 0.72.

^3^Number of observations = 26, the coefficient of determination (*R* ^2^) was 0.405, indicating that the correlation between the observed response value and the predicted value of the response variable was about 0.64.

^4^Number of observations = 27, the coefficient of determination (*R* ^2^) was 0.874, indicating that the correlation between the observed response value and the predicted value of the response variable was about 0.93.

β -CTX: β C-Terminal telopeptide of type I collagen.

## Discussion

In this randomized, controlled trial of daily kefir supplementation in osteoporosis patients with or without osteoporotic fracture, we found that greater short-term treatment-related increases in bone turnover were associated with 6-month increases in BMD. These effects were clearly apparent in those patients with higher baseline serum OC levels and higher total hip or femoral neck BMD as measured by DXA. Higher baseline bone turnover was inconsistently related to 6 month changes in BMD. Our results suggest that patients whose bone turnover levels moved from negative to positive over time derive the greatest densitometric benefit from kefir treatment.

These results are in accordance with studies showing a positive correlation of frequent milk consumption with BMD or bone health [29–31], and that daily supplementation with approximately 700 ml milk decreases bone resorption and improved calcium balance [32]. Studies have repeatedly shown that calcium supplements derived from milk are more effective than those from other sources [30,33]. The reason for this advantage can be the other components in milk, such as a high availability of calcium, an optimal Ca:P ratio of 1.3, the presence of other bone-relevant minerals like magnesium and zinc [16], and bioactive peptides or proteins with the potential to improve calcium absorption and bone mineral density, like CPPs and milk basic protein [18, 34, 35]. This advantage of milk, together with the point in time of milk consumption may explain why supplementation with fermented milk and thus the additional calcium significantly depressed bone turnover in short term treatment. As reported previously, postmenopausal women who daily consumed fermented milk supplemented with calcium, inulin-type fructans and CPPs for two weeks had marked decreases in both (1) nocturnal excretion of deoxypyridinoline, a marker of bone resorption, and (2) bone alkaline phosphatase, a marker of bone formation [24].

Our results also revealed that kefir-fermented milk, when supplemented daily with 1,500 mg CaCO_3_, had no additional beneficial effect on calcium absorption and bone turnover above that of the fermented milk itself. The reason for this might be that the daily doses of 1,500 mg CaCO_3_ were enough to overcome the bone loss in the control group; however, other studies have frequently used doses of 1,000–1,600 mg/d [36–38]. Thus, the supplemented calcium raised the total calcium intake above a threshold level, such that the solubility of luminal calcium was at its limit and no further absorption could occur [39].

Our previous experiments in mice also confirmed that kefir treatment while feeding with CaCO_3_ simultaneously may weaken the effect of increasing bone density of kefir. Several authors have demonstrated that phosphopeptides from tryptic digestion of casein (CPPs) had a high affinity to calcium ions *in vitro* [40,41]. It may be that the alkaline (positive charged) calcium bicarbonate will neutralizing the effect of the acid (negative charged) bioactive peptides in kefir, reducing its potential to improve calcium absorption and BMD.

The serum β-CTX declined over time compared to the baseline value in both groups. Fig 2 showed a decline in the kefir-treated group at the first and the third month after treatment, but β-CTX inversely increased after the sixth month of treatment (Fig 2C). The decreased serum activity of OC decreased significantly in the kefir-treated group compared to the control group after one month of treatment, but gradually increased to levels higher than the control group at three and six months. OC decreased from the beginning of the experiment to six months after treatment (Fig 2D).

Results found in our subjects after one month of intervention with fermented milk as a calcium source is in agreement with long-term reports by others on calcium supplementation in humans [37,42], and on animal models of a diet with a high calcium content [43]. In a previous study in minipigs (an animal model very close to humans, particularly in nutrition physiology), two milk proteins with different potential for CPPs release and their effect on calcium and bone metabolism were studied [44]. Table 2 showed a tendency for higher plasma concentrations of PTH after feeding with a casein-containing diet such as fermented milk [45].

The diminution of OC in context with lower β-CTX levels after a rise in calcium intake is generally assumed to reflect a decrease in bone turnover. The decrease in total serum calcium concentrations during the intervention is difficult to interpret. It may be a secondary effect of the slightly lower serum levels of PTH following the calcium load with the fermented milk. Serum calcium was slightly increased after patients received kefir treatment for six months, but in control subjects, serum calcium was apparent decreased at the sixth month. Serum PTH increased significantly after treatment with kefir, but decreased significantly in the control group. This finding means that bone resorption was decreased in the first month of kefir supplementation and that bone formation started earlier in the intervention than in the control group. PTH may promote bone remodeling after treatment with kefir for 6 months. In other words, taking kefir will help subjects maintain high serum PTH concentrations, which will also enable them to maintain a high calcium concentration.

The effects of kefir consumption on bone were highly variable. However, in terms of both observed changes in bone turnover and subsequent increases in BMD, our data suggest that baseline levels of bone turnover may account for at least some of this variability. Other baseline factors, such as pretreatment BMD, previous fracture history, and recent exposure to antiresorptive agents, may also influence kefir-induced changes in BMD.

However, our data indicate that kefir therapy increases BMD whether or not bone turnover is suppressed. Moreover, and in keeping with the report of Kurland *et al*. [45] on male osteoporosis, we observed that increases in BMD as shown by DXA are, in fact, greater among those with higher baseline procollagen type I N-terminal propeptide (PINP) levels, a marker of bone formation. Similar trends were observed in this study with 1-year changes in spine quantitative computed tomography (QCT) trabecular BMD, although only β-CTX changes reached statistical significance. The strongest and most consistent associations in our study were observed between the 1- and 6-month changes in bone formation markers (particularly OC) and the 6-month changes in total hip and femoral neck BMD. The relationship between bone turnover markers and the change in bone density was greatest and earliest for the bone formation marker OC, for which greater 6-month changes were strongly associated with changes in total hip and femoral neck BMD.

Conversely, the changes in bone resorption, as measured by β-CTX, were most apparently decreased in subjects with T-scores greater than -1 after 3 months of kefir treatment. These observations support the hypothesis that kefir increases bone mass initially by decreasing bone resorption first and augmenting osteoblast function earlier than in those who do not take it.

These findings are likely to be attributable to the faster rate of bone remodeling and greater response to kefir in treated subjects. The association between 1-month changes in markers and the 6-month change in BMD also tended to be stronger with bone formation markers, especially OC, than with β-CTX, a sensitive bone resorption marker. Thus, because of its rapid and marked increased after kefir administration, serum OC may be the single most sensitive biochemical marker with which to monitor kefir efficacy.

Our study is short-term and therefore, by design, does not include fracture outcome data. As shown in Tables 1–4, the regression coefficient estimates and the associated standard error estimates of the kefir treatment and the other covariates found in this pilot study could assist in the design of an appropriately powered study in future. Additional studies are needed to determine the relationship of 6-month changes in DXA to long-term fracture outcomes. Because we studied osteoporosis patients at high risk of fracture, our results may not apply to other populations.

## Conclusions

In summary, in this study of bone turnover markers and changes in BMD in osteoporosis patients treated with kefir-fermented milk, we found that baseline bone turnover was associated with subsequent changes in BMD. Short-term treatment-related changes in bone turnover markers, especially bone formation, were strongly associated with subsequent changes in BMD. Our results suggest that serial measurement of bone turnover shortly after initiation of kefir therapy may be helpful in assessing the ultimate therapeutic response to kefir-fermented milk.

## Supporting Information

S1 Appendix IRB approval certificate from the Institutional Review Board of Tri-Service General Hospital Medical Center (TSGH-IRB TC098-13). (PDF)

S2 Appendix Clinical trial registration document from the ClinicalTrials.gov (Identifier: NCT02361372). (PDF)

S3 Appendix Clinical trial protocol in Chinese and English version. (PDF)

S4 Appendix CONSORT checklist of information for the report of a randomized trial. (DOCX)
