# Supplementary material for: Short-Term Effects of Kefir-Fermented Milk Consumption on Bone Mineral Density and Bone Metabolism in a Randomized Clinical Trial of Osteoporotic Patients
Source: PLoS One. 2015 Dec 10;10(12):e0144231. doi: 10.1371/journal.pone.0144231 (PMC4675497; doi:10.1371/journal.pone.0144231)
Supplement: S1 Appendix — (PDF) [file pone.0144231.s001.pdf]

Tel : 886-2-87923311 ext 10552
Fax: 886-2-2793-6049
E-mail: tsghirb@ndmctsgh.edu.tw

Institutional Review Board of
Tri-Service General Hospital
National Defense Medical Center

No.325, Sec.2, Cheng-Kung Rd. Neihu
11490, Taipei, Taiwan, R.O.C

Letter of Approval

Date : Mar-19-2010
Protocol Title : The study in fermented milk treating for osteoporotic patients
Protocol number: TC098-13
Protocol version: version date : 10-Mar-2010
ICF version: version date : 10-Mar-2010
Chinese Abstract version: version date : 10-Mar-2010
Principal Investigator : Min-Yu Tu
Co-Principal Investigator : Chuan-Mu Chen
TSGHIRB Approval Number: TC098-13
Expiration Date : Mar-18-2011

On Mar-19-2010, the Institutional Review Board of the Tri-Service General Hospital, National Defense Medical Center (TSGHIRB) approved the above named Protocol, Subject Information/Consent Form, and related materials **as low risk in classification, report frequency 1 time/year.**

Your ongoing responsibility to the TSGHIRB follows:

- any changes to the approved protocol and/or related materials submit for IRB approval before instituting these changes. Changes to eliminate immediate hazards to human subjects are the only changes that may be made prior to IRB approval.
- submit a protocol status update report for continuing review.
- submit a protocol status update report with your findings to the IRB office when your protocol is completed or terminated.
- report all serious and unanticipated adverse events in studies involving human subjects to the IRB immediately.
- use only copies of the enclosed subject information/consent form, flyers, etc. that bear the TSGHIRB approval date stamp.

If you have questions, concerns, or requests for more information , please contact TSGHIRB office at 886-2-8792-3311 ext.10552.

<u>Please reference your TSGHIRB approval number in all communications about this protocol.</u>

Sincerely,
Pauling Chu, M.D., Ph.D.

Pauling Chu

Chairman

三軍總醫院 TSGHIRB 人體試驗審議委員會

TEL: 886-2-87923311 ext 10552
FAX:886-2-2793-6049 / 2792-9201
E-mail: tsghirb@ndmctsgh.edu.tw

國防醫學院三軍總醫院
人體試驗審議會

11490 台北市內湖區成功路二段
325 號 醫療大樓五樓 5113 室
No. 325, Sec.2, Cheng-Kung Rd.
Neihu 11490, Taipei, Taiwan, R.O.C

人體試驗計畫同意函

查國軍台中總醫院杜旻育醫師主持之「發酵乳治療骨質疏鬆症患者效果之研究」(TSGHIRB 核准編號：TC098-13）研究計畫案持續審查報告，業經本院 2015 年 2 月 25 日人體試驗審議會第一審議會審查決議通過，並同意繼續執行，該計畫案經評估屬低度風險，**(持續審查頻率為每年一次)**，有效期限至 2016 年 2 月 24 日，特此證明。

計畫主持人應於同意函有效期屆滿前一個月提出展延申請，本案須經本院人體試驗審議會通過後，方可繼續執行。

人體試驗審議會召集人

余慕賢

Letter of Approval

Tri-Service General Hospital National Defense Medical Center

Date of approval: 02/25/2015

TSGHIRB No.：TC098-13

Protocol Title：The study in fermented milk treating for osteoporotic patients

Principle Investigator：Dr. Min-Yu Tu

Sub Investigator：Chuan-Mu Chen

On 02/25/2015, the Institutional Review Board I of the Tri-Service General Hospital, National Defense Medical Center decided to approve the above-named application. According to the written operating procedures, GCP, and the applicable regulatory requirements, this application is approved by the Institutional Review Board of TSGHIRB. The board is organized under, and operates per International Conference on Harmonization (ICH) / WHO Good Clinical Practice (GCP) and the applicable laws and regulations.

This approval is valid for **1 year** till **02/24/2016** The principle investigator is required to submit the application for extension 1 month before the expiration date.

Sincerely,

Mu-Hsien Yu,M.D., Ph.D.

Chairman

Institutional Review Board

The Committee is Organized and operates in accordance with ICH6 GCP regulations and guideline.
本審議會組織與運作皆遵守ICH6 GCP規定

Tel : 886-2-87923311 ext 10552
Fax: 886-2-2793-6049
E-mail: tsghirb@ndmctsgh.edu.tw

**Institutional Review Board of
Tri-Service General Hospital
National Defense Medical Center**

No.325, Sec.2, Cheng-Kung Rd. Neihu
11490, Taipei, Taiwan, R.O.C

Letter of Approval

Date : May-28-2011
Protocol Title : The study in fermented milk treating for osteoporotic patients
Protocol number: TC098-13
Protocol version: version date : 10-Mar-2010
ICF version: version date : 10-Mar-2010
Chinese Abstract version: version date : 10-Mar-2010
Principal Investigator : Min-Yu Tu
Co-Principal Investigator : Chuan-Mu Chen
TSGHIRB Approval Number: TC098-13
Expiration Date : Mar-18-2012

On May-28-2011, the Institutional Review Board of the Tri-Service General Hospital, National Defense Medical Center (TSGHIRB) approved the above named Protocol, Subject Information/Consent Form, and related materials **as low risk in classification, report frequency 1 time/year.**

Your ongoing responsibility to the TSGHIRB follows:

- any changes to the approved protocol and/or related materials submit for IRB approval before instituting these changes. Changes to eliminate immediate hazards to human subjects are the only changes that may be made prior to IRB approval.
- submit a protocol status update report for continuing review.
- submit a protocol status update report with your findings to the IRB office when your protocol is completed or terminated.
- report all serious and unanticipated adverse events in studies involving human subjects to the IRB immediately.
- use only copies of the enclosed subject information/consent form, flyers, etc. that bear the TSGHIRB approval date stamp.

If you have questions, concerns, or requests for more information , please contact TSGHIRB office at 886-2-8792-3311 ext.10552.

<u>Please reference your TSGHIRB approval number in all communications about this protocol.</u>

Sincerely,
Pauling Chu, M.D., Ph.D.

Chairman
