# Supplementary material for: Short-Term Effects of Kefir-Fermented Milk Consumption on Bone Mineral Density and Bone Metabolism in a Randomized Clinical Trial of Osteoporotic Patients
Source: PLoS One. 2015 Dec 10;10(12):e0144231. doi: 10.1371/journal.pone.0144231 (PMC4675497; doi:10.1371/journal.pone.0144231)
Supplement: S2 Appendix — (PDF) [file pone.0144231.s002.pdf]

ClinicalTrials.gov PRS DRAFT Receipt (Working Version)
Last Update: 08/30/2015 21:21

Kefir on Bone Mineral Density and Bone Metabolism in Osteoporotic Patients

This study has been completed.

Sponsor:	Ministry of Science and Technology, Taiwan
Collaborators:	
Information provided by (Responsible Party):	Min-Yu Tu, Ministry of Science and Technology, Taiwan
ClinicalTrials.gov Identifier:	NCT02361372

Purpose

In a controlled, parallel, double-blind intervention study over 6 months, the investigators investigated the effects of kefir-fermented milk (1,600 mg/kg) supplemented with calcium bicarbonate (CaCO3, 1,500 mg/kg) and bone metabolism in 40 osteoporosis patients, and compared them with CaCO3 alone without kefir supplements. Bone turnover markers were measured in fasting blood samples collected before therapy and at 1, 3, and 6 months. BMD values at the spine, total hip, and hip femoral neck were assessed by dual-energy x-ray absorptiometry (DXA) at baseline and at 6 months.

Condition	Intervention	Phase
Osteoporosis	Placebo<br>CaCO3<br>Kefir	N/A

Study Type: Interventional
Study Design: Treatment, Parallel Assignment, Double Blind (Subject, Investigator), Randomized, Efficacy Study
Official Title: Short-term Effect of Kefir-fermented Milk Consumption on Bone Mineral Density and Bone Metabolism in Osteoporotic Patients

Further study details as provided by Min-Yu Tu, Ministry of Science and Technology, Taiwan:
Primary Outcome Measure:
- bone mineral density and bone regeneration [Time Frame: 3 months] [Designated as safety issue: Yes]

Enrollment: 69
Study Start Date: May 2010
Primary Completion Date: January 2012
Study Completion Date: January 2012

Arms	Assigned Interventions
Experimental: Kefir and CaCO3<br>Kefir were administered 1,600 mg kefir-fermented milk per day and an	Kefir<br>Kefir daily for 6 months

Arms	Assigned Interventions
accompanying supplement of 1,500 mg CaCO3 for 6 months	
Placebo Comparator: Placebo and CaCO3<br>Placebo and 1,500 mg of CaCO3 daily for 6 months	Placebo<br>Placebo daily for 6 months<br>CaCO3<br>CaCO3 daily for 6 months

Eligibility

Ages Eligible for Study: 55 Years to 70 Years
Genders Eligible for Study: Both
Accepts Healthy Volunteers: Yes

Criteria

Inclusion Criteria:

- Clinical diagnosis of osteoporosis patients

Exclusion Criteria:

- Any previous use of parathyroid hormone or sodium fluoride, use of anabolic steroids or growth hormone within 6 months before trial entry or oral or intravenous systemic corticosteroids within 12 months, and any previous use of strontium.

More Information

Responsible Party:	Min-Yu Tu, Ministry of Science and Technology, Ministry of Science and Technology, Taiwan
Study ID Numbers:	NSC-99-2324-B-005-017-CC1
Health Authority:	Taiwan: National Science Council

U.S. National Library of Medicine | U.S. National Institutes of Health | U.S. Department of Health & Human Services
