# Supplementary material for: Short-Term Effects of Kefir-Fermented Milk Consumption on Bone Mineral Density and Bone Metabolism in a Randomized Clinical Trial of Osteoporotic Patients
Source: PLoS One. 2015 Dec 10;10(12):e0144231. doi: 10.1371/journal.pone.0144231 (PMC4675497; doi:10.1371/journal.pone.0144231)
Supplement: S3 Appendix — (PDF) [file pone.0144231.s003.pdf]

國防醫學院三軍總醫院人體試驗審議委員會
人體試驗計畫申請書

（本申請書字體不得小於 14 號字，含計畫主持人簽名頁及附表共　　頁）

申請日期:　99　年 01 月 01　日

1.試驗名稱(中文)：發酵乳治療骨質疏鬆症患者效果之研究

試驗名稱(英文)：The study in fermented milk treating for osteoporotic patients

2.計畫主持人姓名(中文)：　杜旻育　　　　　　(英文)：Min-Yu TU

單位：國軍台中總醫院骨科

職稱：主治醫師

聯絡電話： 04-23934191-525368　　　E-mail：du0807@yahoo.com.tw

臨床試驗相關訓練證明時數：近三年內共 14 小時

計畫協同主持人姓名(中文)： 陳 全 木　　　　(英文)：Chuan-Mu Chen

單位：國立中興大學生命科學系

職稱：特聘教授兼研發長

聯絡電話：04-22840319-701　　E-mail：chchen1@dragon.nchu.edu.tw

臨床試驗相關訓練證明：　☐有(請檢附)　　☐無

3.預計參與試驗計畫計 1 家醫院，本院協同主持人及其他醫院/單位計畫主持人(請依人數增列)

姓　名	單　位	職　稱
杜旻育	骨科	主治醫師

4.計畫主持人目前參與試驗計畫 0 件，目前收錄受試者人數 0 位，參與之研究人員 1 位，參與之協同主持人 1 位。

5.試驗類別：

☐01 新藥品(☐查驗登記☐學術研究)　　☐02PK 或 BA/BE
☐03 新醫療器材　　☐04 新醫療技術
☑05 發表論文/一般學術研究

6.計畫性質(複選)：

☐多國多中心　☐本國多中心　☑本國單一中心
☐基因相關臨床試驗　☐上市後監測調查(PMS)

□其他(請註明:______________________)
□如為社區研究，請說明本研究對社區的影響與協商過程：

7.試驗品項簡介(請檢附附表)：

□藥品（格式如附表一）
□疫苗（格式如附表二）
□基因研究（格式如附表三）
□醫療器材（格式如附表四）
□醫療技術（格式如附表五）
☑其他：□問卷　□病歷回顧　□社會行為科學研究☑請註明：_發酵乳食品_

8.試驗計畫經費來源

☑委託廠商單位__中國化學製藥股份有限公司_____
☑學術研究(□國科會□衛生署□國衛院□本院民診基金□中研院☑其他_________)
□自籌(自行研究無獲得經費補助)
請填列計畫預算總經費為：　　　元

9.試驗階段(目的)

□phase I　了解藥物毒性為目的之安全性研究
□phase II　了解療效為目的之初步療效觀察，對象為病人
□phase III　完整療效評估，對象為病人
□phase IV　上市後研究
☑其他　　觀察研究

10.登錄於 ClinicalTrials.gov 網站之 Identifier：杜旻育

或 其他登錄網站及其 Identifier：

11.須審查本計畫結果之主管機關

□行政院衛生署	□美國食品藥物管理局(FDA)
□歐洲檢驗藥品局(EMEA)	□日本厚生勞動省
☑學術發表	□其他(請註明_______________)

12.試驗內容簡介

12.1 試驗產品/藥品名稱（學名、商品名、規格）:(□不適用）

發酵乳克弗爾 (Kefir) 緣起於數百年前之北高加索區，係由克弗爾菌元－克弗爾粒 (Kefir grain)發酵各類乳品而成

12.2 研究設計：　□平行研究　□交叉研究　☑其他設計

12.3 組數：　□單組　☑雙組　□多組：_____組

12.4 樣本數：本院__90___人(全球人數________人國內________人)；接受有效治療人數比率：__100___%

12.5 病患接受追蹤期間：____24___ 周(短期追蹤請填______天)

12.6 是否有： ☑隨機分配 ☑對照組
☐期中分析（mid-term analysis）
☐資料安全委員會(DSMB, Data Safety Monitoring Board)
☐主持人手冊

12.7 研究起迄期間： 99 年 4 月 1 日 至 100 年 3 月 31 日

12.8 計畫實施機構：☐國防醫學院☐三軍總醫院☑其他：國軍台中總醫院

(依國防醫學院條例國防醫學院學生不得參加臨床試驗)

執行地點：☐本院臨床試驗中心 GCRC(請先與 GCRC 聯繫)
☑門診處(請說明何處)__骨科門診__
☑病房(請說明何處)__骨科病房__
☑其他(請說明何處)__骨科治療室__

12.9 計畫是否使用藥物：☑否 ☐是

12.10 是否使用安慰劑試驗？ ☑否 ☐是

12.11 本試驗過程中受試者將接受輻射處理 ？ ☐否 ☑是

(若☑是請續填下列資料)

輻射情況(X 光或核子放射線)

使用輻射種類、方法 總 次 數

計畫過程中是否利用輻射化學物質在人體進行試驗？

☑否 ☐是(請說明何種物質)__________

本計畫受試者接受輻射處理，是否屬於常規性診斷或治療手續？

☐否 ☑是

13. 個案報告表

☑檢附個案報告表(臨床試驗計畫必須檢附)

☐免除個案報告表

請說明理由：

14.招募病患方式(請勾選)

☑計畫主持人(含共/協同主持人)口頭介紹

☑其他醫師護士 (非共/協同主持人)口頭介紹
☐海報資料(是否已附上：☐否 ☐是)
☐網路資料(是否已附上：☐否 ☐是)
☐其他(請說明)________________

15.受試者同意書檢查項目

15.1☑檢附受試者同意書

☐免簽署受試者同意書（申請此項案件不得快速審查，若委員會未核准需補附重新審查）

請說明理由：

15.2 由誰向受試者或其法定代理人解釋試驗內容並取得同意(請說明)？

醫師及研究助理

15.3 取得同意的時間？☑篩選前 ☐篩選後，隨機分派前

15.4 在什麼地點解釋試驗內容及何處獲得本受試者同意書(請寫明取得地點，例何處診間或病房)？每件約花費多久時間？(請說明)

骨科門診或病房由醫師評估說明解釋並取得受試者同意書。每件約花費30 分鐘

15.5 除了簽署受試者同意書以外，如何確保受試者或其法定代理人對試驗內容了解？

☑與受試者及其家人共同討論
☐與受試者及協助說明者共同討論
☐另安排時間作追蹤
☐其他（請說明）____________________

15.6 本計畫是否納入易受傷害團體為受試者？

☑ 否☐ 是，

如果☑是，請勾選下列項目：

☐兒童（未達法定年齡不得簽署同意書者）
☐小於 7 歲 ☐8~19 歲
☐孕婦／新生兒 ☐受刑人 ☐重症末期病人
☐其他__________________________

15.7 是否將支付受試者酬金 ☑否 ☐是，金額______________

15.8 取樣是否抽血？ ☐否☑是(如果☑是，請填寫下列項目)

每次抽血量 10 CC/次，抽血次數 2 次

計畫主持人聲明書

(執行本計畫之主持人、共同主持人皆須簽署此聲明)

一、本人明瞭人體試驗之執行應符合赫爾辛基宣言的倫理原則，並願依赫爾辛基宣言的精神及國內相關法令的規定，確保試驗對象之生命、健康、個人隱私及尊嚴。

二、本人承諾試驗計畫應依照主管機關或人體試驗審議委員會核准之試驗計畫書執行。

三、本人明瞭並遵守優良藥品臨床試驗規範和衛生署相關的法規要求，及接受相關主管機關的查核。

四、本人明瞭試驗主持人應完全熟悉試驗藥品於試驗計畫書、最新版主持人手冊、驗藥品／醫療器材或醫療技術資訊，及其他由試驗委託者提供之產品資訊中描述之使用方法。

五、本人承諾確保所有協助人體試驗的相關人員對試驗計畫書及試驗藥品／醫療器材或醫療技術有充分的了解，以及他們在人體試驗中相關的責任和工作。

六、本人承諾所有人體試驗資料應予紀錄、處理、建檔及存檔管理，以供確實報告、呈現及確認。

七、計畫主持人應負責所有人體試驗相關的醫療決定。

八、在受試者參加試驗與後續追蹤期間，本人會確保對受試者任何與試驗相關的不良反應，包括重要實驗室檢查值等，提供充分的醫療照護。當計畫主持人察覺試驗期間受試者有疾病需要醫療照護時，必須告知受試者。

九、本人承諾依國內相關法令的規定通報嚴重不良反應事件及繳交期中及結案報告，並提供計畫進行中任何影響受試者安全及權益等資訊給三軍總醫院人體試驗審議委員會，以作確保受試者權益之審核。

十、本人明瞭三軍總醫院人體試驗審議委員會及主管機關得採取必要因應之追蹤審查及措施並得調閱相關資料，必要時得要求中止或終止該計畫。

十一、本人承諾計畫進行中或結束後均須保護受試者隱私，且研究計畫需維護可辨識資料之機密性，符合相關法規對隱私及機密之規定。

十二、本人承諾遵循所提出之簽署受試者同意書程序，並由本人或授權之團隊成員完整詳細的解說並取得知情同意。試驗執行前，應獲得受試者自願給予之受試者同意書。執行時應確認使用有蓋上本委員會核准章之最新版本受試者同意書。

十三、若計畫執行之內容有所變動，除了要立即降低危險性的情況外，在未獲得三軍總醫院人體試驗審議委員會同意前，絕不會進行修改後的內容。

十四、試驗中止或終止時，應立即通知主管機關及三軍總醫院人體試驗審議委員會，並確保受試者有適當之治療及追蹤。

十五、本人切結，三年內未受醫師法第 25 條醫師懲戒之確定處分。

十六、本人切結，三年內未曾於擔任試驗主持人時，因重大或持續違反 GCP 者。

計畫主持人中文正楷姓名： 杜旻育　　簽名: 杜旻育

日期: 2010, Jan. 1.

共同主持人中文正楷姓名：陳全木　　簽名: 陳全木

日期: 2010, Jan. 1.

(共同主持人請依人數需要自行增減欄位)

Institutional Review Board of Tri-Service General Hospital National Defense Medical Center

Human Trail Program Application

Application Date: 2010 January 1st

1. Trial Program: The Study in fermented milk treating for osteoporotic patients
2. Program Moderator: Min-Yu Tu

   Service Unit: Taichung Armed Forces General Hospital

   Title: Physician

   Tel: 04-23934191-525368 Email: du0807@yahoo.com.tw

   Proof of Associated human trial training hours: Within 3 years : 14 hours

   Program Assisting Moderator: Chuan-Mu Chen

   Service Unit: National Chung Hsing University, Department of Life Science.

   Title: Distinguished Professor and Develop Executor

   Tel: 04-22840319-701 Email: chchen1@dragon.nchu.edu.tw

   Proof of Associated hum trial training hours: ☐Yes (Please provide Proof)

   ☐ No
3. The Attendees of Hospital 1 hospital. Attendees of personnel from current hospital or other hospital for Program Moderator or assisting Program Moderator: (Please Name All below)

Name	Unit	Title
Min-Yu Tu	Orthopedics	Physician

4. Trial program attending for program Moderator 0 . Trial program volunteers 0. Trial program researcher 1. Program Assisting Moderator 1.
5. Trial Category

   ☐01 New Medicine ☐02 PK or BA/BE

   ☐03 New Medical Device ☐04 New Medical Technique

   ☑05 Published Paper/Medical Research
6. Program Characteristic

   ☐Multi Country/Multi Center ☐One Country/Multi Center

   ☑Once Country/One Center ☐Gene relative Human Trial

   ☐PMS (Marketing Monitor and research)

   ☐Other (Please specify:______________)

   ☐If applicable to community research please specify the influence to the

community due to the research and negotiation process with the community.

7. Trial Items Summary

☐Medicine (Please refer to Attachment No.1)

☐Vaccine (Please refer to Attachment No.2)

☐Gene Research (Please refer to Attachment No.3)

☐Medical Device (Please refer to Attachment No.4)

☐Medical Technique (Please refer to Attachment No.5)

☑Other: ☐Questionnaire ☐Retrospective ☐Social Behavior Sciences and Research ☑Please Specify: Fermented milk food.

8. Trial Research Funds

☑Entrust Company: Chunghwa Chemical & Pharmaceutical Co., Ltd.

☑Academic Research(☐NSC ☐Department of Health ☐National Institutes of Health ☐Hospital Clinic Funds ☐Academia Sinica ☑Others________)

☐Self funding (Stand alone Research without any funding)

Please specify funding budget: ______ NTD

9. Trial Phase (Purpose)

☐Phase I Research on the safety of toxic level in Medicine.

☐Phase II Research on the efficacy after initial treatment. Patient Observation.

☐Phase III Research on the efficacy after treatment. Patient observation.

☐Phase IV After Market Research.

☑Others Observation Research.

10. Identifier is register on ClinicalTrails.gov Min-Yu Tu or on other registered website.

11. Authorities Examining Program Result

☐Department of health ☐FDA

☐EMEA ☐Japanese Ministry of Health

☑Academic Publication ☐Others (Specify:___________)

12. Trial Contents Summary

12.1 Trial Products/Medicine (Scientific Name, Product Name, Specs): (☐Not applicable)

Kefir Fermented milk is discovered hundred years ago in northern part of Caucasus. Made with Kefir Grain ferment with different type of milk.

12.2 Design of Research ☐Parallel Study ☐Crossover Study ☑Other design.

12.3 Number of Groups: ☐Single ☑Double ☐Multi:____ Groups

12.4 Number of Samples: Domestic Hospital 90 patient (World count ____Patient.

Domestic____Patient) Accepted effective treatment patient ratio: 100%.

12.5 Patient Tracing Period:__24__Weeks (Short term tracing ___days)

12.6 Presence of:

☑Randomly assigned ☑Control Group

☐Mid Term Analysis

☐DSMB, Data safety monitoring Board)

☐ Moderator Manual.

12.7 Research Period: 2010 April 1st to 2011 March 31st

12.8 Agency for the Program execution: ☐National Defense Medical Center ☐Tri-Service General Hospital ☑Other: Taichung Armed Forces General Hospital (Due to the restriction of National Defense Medical Center, Students are not allowed to participate in Human Trials)

Place of execution:

☐Clinical Trail Center located in the Hospital (Please contact GCRC prior to the trail)

☑Outpatient Department. Specify: Orthopedic OPD

☑Ward Area. Specify: Orthopedic Ward.

☑Others. Specify: Orthopedic Treatment Room

12.9 Medication used with this Program: ☑No ☐Yes

12.10 Use of Placebo: ☑No ☐Yes

12.11 During the process of this program, is the contestant going under exposure of radiation? ☐No ☑Yes

(If yes, please filled out the questionare below)

Radiation Situation (X Ray or MRI)

Radiation Type and usage   Total Usage by times

During the program, is there usage of radiation substance for the human trials?

☑No ☐Yes please specify the substance________

The Contestant in this program, who is going under radiation, is in Regular therapy or processes needed for examining?

☐No ☑Yes

13. Case Report Criteria

☑Case Report Data Sheet (Human Trial Program Need to supply)

☐Without Case report data sheet

Please explain:

14. Recruiting Patients:

☑Program Moderator, (including co- Moderator and or assisting Moderator) with verbal introduction

☑Nurse or Other Physician (Non co- Moderator and or assisting Moderator) with verbal introduction

☐Poster

☐Internet

☐Other (explain)____________

15. Contestant Agreement Checking Criteria

15.1 ☑Contestant Agreement

☐Contestant Agreement free (Appling for this program can not be under fast track application. If committees disagreed resubmission of the paper work is needed.)

Please explain:

15.2 The personnel for explaining the full process to the contestant or the legal representative and obtain the agreement? (please explain)

Physician and Assistant researcher

15.3 The time frame for obtain the agreement:

☑ Prior to screening

☐ After screening or prior to random assign

15.4 The place for explaining the full process and obtaining the agreement. (Please write down the place like OPD or at the ward) And the time used for each case. (Please explain)

In Orthopedic OPD or Orthopedic Ward, explained by physician and obtain the agreement. Used proximately 30 min per each contestant.

15.5 How do the contestant or the legal representatives understand the process besides the information given through the agreement?

☑Consulting with the contestant and the family members

☐Consulting with the contestant and legal representatives

☐Arrange another time for follow up.

☐Others (Explain)___________

15.6 This program includes vulnerable groups as contestant?

☑No ☐Yes,

If yes, please select the following

☐ Child (The agreement can not be signed if the age is under the legal age.)

authorities have taken the necessary response to the review and measures to track and have access to relevant information. If necessary, may request with suspension or termination of the program.

11. Regarding to the provisions of relevant laws of privacy and confidentiality, the contestant must be protected with privacy protection prior, during and post of the program. Research projects required to maintain the confidentiality and the information can be identified.
12. The Moderator or the Authorized team members are promised to follow the procedure to fully explained and obtained the agreement signed by the contestant. Prior to the program, all contestants shall submit the agreement voluntarily. The latest or updated agreement with approval from the commission shall be submitted and confirmed when proceeding with the program.
13. The program content can not be modified, except reducing the risk of the program, without obtaining the agreement by the TSGH Human Trail Committee.
14. Notify the competent authorities and TSGG Human Trials Committee immediately when the program is suspended or terminated. This is to ensure the contestant to receive proper treatment and tracking.
15. Respect to the Physician Law Article 25, Moderator is not charged with any disciplinary sanctions within 3 years.
16. Moderator did not due to gross or persistent violation by GCP while being a moderator within 3 years.

Moderator Signature (Print): Min-Yu Tu Sign: Min-Yu Tu

Date: 2010. Jan. 1

Co-Moderator Signature (Print): Chuan-Mu Chen Sign: [signature]

Date: 2010, Jan. 1.

(Please adjust the space if there's more Co-Moderator)

國軍臺中總院醫學研究發展計畫申請表

研究計畫編號：____________________________________

研究起迄年：自 2010 年起至 2012 年止

研究計畫名稱：

中　　文：發酵乳治療骨質疏鬆症患者效果之研究

英　　文：The study in fermented milk treating for osteoporotic patients

研究計畫重點：發酵乳治療骨質疏鬆性症患者之療效優於或等同於其他骨質疏鬆之藥物

申 請 單 位 ：國軍 台中 醫院 ________ 部 骨 科

申　請　人：杜旻育

國 軍 臺 中 總 院 醫 學 發 展 計 畫 書

壹、綜合資料：　　　　　　　　　　　　　　　　　　　　計畫編號：(務必填寫)

申請單位	骨科		執行單位	骨科，中興大學生科系	
計劃主持人	杜旻育		協同主持人	陳全木教授兼系主任	
執行期限	本年度計劃：自至 自2010年5月1日起至2012年4月31日止 起止		全程計畫：自至 自2010年5 月1日起至2012年4月31日止 起止		
計畫名稱	中文：發酵乳治療骨質疏鬆症患者效果之研究<br>英文： The study in fermented milk treating for osteoporotic patients				
計畫歸屬	(　　)野戰衛勤	(ˇ)基礎、預防醫學	(　　)選兵醫學		
	(　　)戰傷醫學	(　　)航太醫學	(　　)潛水醫學		
	(　　)核生化防護	(　　)臨床醫學	(　　)其他		
研究計劃預算	項　目	金　額	說　明		
	人事費	100,000	計畫主持費及研究助理費		
	事務費	30,000	影印書籍論文		
	業務費	20000	IRB 申請費及受訓相關報名費		
	維護費				
	旅運費				
	材料費	450,000	DEXA 約 200000 病人其他生化檢驗 250000		
	設備及投資				
	其　他				
	合　計	600,000	100%		
計畫聯絡人	姓　名：杜旻育　　電　話：04-23935823<br>地　址：台中縣太平市中山路二段348號				

貳、計畫摘要（請於五百字內就計畫要點作一概述，並分別以中英文列出關鍵詞）：

中文摘要

計畫目的：

隨著老年人口的逐漸增加，骨質疏鬆症成為一個重要的健康問題。隨著歲月流逝，骨質將逐漸流失，而發生所謂的骨質疏鬆症 (Osteoporosis)；當骨質流失後的骨骼受到外力衝擊時，便容易因結構支撐力不足而脆裂，因而發生骨折。發酵乳克弗爾 (Kefir) 緣起於數百年前之北高加索區，係由克弗爾菌元–克弗爾粒 (Kefir grain)發酵各類乳品而成，除具有一般發酵乳風味外，更含有微量酒精與二氧化碳所產生之獨特口感，具有乳香檳之稱。克弗爾菌元由乳酸菌和酵母菌共組共生菌相(symbiotic yeast-bacteria community)，因氣候、培養條件及乳原料不同，各區菌元組成有異。目前許多國家皆有克弗爾相關商品上市。而克弗爾發酵乳的保健機能性的相關研究也多被證實，包括腸內菌相、提升免疫力、抗腫瘤活性、降膽固醇等。此研究將以發酵乳治療骨質疏鬆症患者，治療三及六個月之後，以雙能量式 X 光骨質密度檢查 (DEXA)對其功效進一步評估。

實施方法：

被診斷為骨質疏鬆症病患分為兩組：

發酵乳治療組

病患分為兩種劑量 每天須服用 1600mg 及 2400mg 之發酵乳，並補充 1500mg 之鈣質及補充 400 至 800 單位的維生素 D

CONTROL 組

病患只補充 1500mg 之鈣質及補充 400 至 800 單位的維生素 D

臨床評估

1.以雙能量式 X 光骨質密度檢查 (DEXA)評估病人治療前後三個月及半年後病患骨質密度改善程度。

2. 病人治療前及治療半年後，接受抽血約 10cc 兩次共計 20cc。

生化檢驗:如血中之鈣磷濃度、副甲狀腺素(PTH)、Osteocalcin、Deoxy-pyridnoline cross-links、**bone turnover markers..等**。

關 鍵 詞：骨質疏鬆、發酵乳克弗爾 (Kefir)、雙能量式 X 光骨質密度檢查 (DEXA)

英文摘要：

Osteoporosis is an important healthy problem in increasing elder population. Bone loss increasing years by years. It happed we called “osteoporosis”. Loss of calcium and phosphorous from bone will result in osteoporosis and after bone loss it will get to easy bony fracture after trauma. Fermented milk (Kefir) was original from North Caucasian area hundreds years ago. It produced from microbial community of Kefir grain fermented kids of milk. It owns not only general fermented milk flavor but also has unit texture with alcohol and carbon dioxide. It is also called milky champagne. The symbiotic yeast-bacteria community of Kefir grain contained Lactobacillus and Saccharomyces. The symbiotic yeast-bacteria community of Kefir is different due to different weathers, culture conditions and original milk. Many countries have Kefir grain fermented milk products available in the markets. The fermented milk study in healthy protection had published including intestine bacteria community, increased immunity, decreased cholesterol, …etc. This study will treat osteoporosis patients with fermented milk for receiving three to six months later. Evaluate the efficiency in osteoporosis by following bone mass with dual-energy X-ray absorptiometry (DEXA) and osteoporosis-associated biochemistry tilter.

Key words: <u>osteoporosis, fermented milk (Kefir) , dual-energy X-ray absorptiometry (DEXA)</u>

參、計畫內容

一、計畫緣起：請描述本計畫產生之背景及其重要性，並包含下列必備資料：<br>1.政策或法令依據。2.問題狀況及發展需求。3.國內、外相關研究之文獻探討<br>4.本計畫與衛生醫療保健之相關性…………等。

由於現代醫療技術發展進步，民眾生活水準和經濟情況的改善，人們享有更舒適與方便的生活，但也因此身體勞動量大幅減少，骨質疏鬆症變成一項流行病症。骨質疏鬆症(Osteoporosis)是慢性病的一種，又稱為「無聲無息的流行病」或「寂靜之疾病」，患得此病通常是在不知不覺的情況下，等到發生骨折或引起其它合併症後才明白罹患此病。而世界衛生組織曾宣稱骨質疏鬆症是僅次於心臟血管疾病的第二大難題，並特別提醒這是一個常受忽視、且診斷不足的疾病，各國都必須將該疾病認定是一個應該提高警覺的重大公共衛生問題。目前老年人口的增加，骨質疏鬆症的預防亦將成為一個重要的課題。目前大都是以測定骨質密度觀察骨質流失情況，如果是危險族群，則在 30 歲以前就必須多補充鈣質，運動..等保存骨本，所謂保健勝於治療，做好預防措施，能建立堅硬骨骼之目的，以降低疾病的產生，以提升生活品質及減輕家人照護及降低醫療支付的負擔。

骨質疏鬆症定義

質疏鬆症是:低密度以及骨組織微細結構受到了破壞，造成骨頭較脆弱，導致骨折發生的危險性增加的一種疾病(osteoporosis is a skeletal characterized by low bone mass and microchitectural with a resulting increase in bone fragility and hence susceptibility to fracture)[1]。骨質疏鬆症的意思就是佈滿了空孔隙的骨骼(圖 2-1)。孔隙多的骨骼和正常骨骼相比較，外型是一樣，但質量卻減少了，其原因為骨頭裡的鈣質逐漸流失，使得內部骨質變為單薄，造成許多孔隙，呈現中空疏鬆的現象，簡言之，由於較低的骨量和骨顯微結構的破壞，而造成骨的脆性增加，以及間接導致骨折機會增加的一種疾病。骨質密度是決定骨質疏鬆症重要的決定因子之一[2]，但是其他因子例如：是否容易跌倒[3] 、骨頭質量和骨頭結構亦是影響因素之一[4] ，世界衛生組織定義:骨質疏鬆症為骨質密度小於年輕健康成人平均值 2.5 個標準差以下者(WHO ,1994)。(圖 2-2)

骨的再塑過程

正常人的骨骼會不斷地進行增生與修復，而成熟的骨骼是維持在一動態的狀平衡下，不斷地由蝕骨細胞與造骨細胞在骨小樑的表面與哈維氏系統內進行破壞與修復的功能。即不斷地進行一連串由蝕骨細胞與造骨細胞重複進行的骨吸收與骨形成作用，稱為骨再塑作程(Bone remodeling)(圖 2-3)。

骨質疏鬆症的致病機轉

骨骼的骨質自出生後會隨著年紀而增加，約 30 歲達高峰(圖 2-4)，不過，骨骼成長的的正向時期只持續到 35 歲。35 歲是骨骼發育的分水嶺，35 歲以後如果收入大於支出，即可得到正平衡；反之，如果持續支出而缺乏補給，就會處於虧損狀態，使骨質不斷流失[5]。很多環境因子與骨質調節有關，包括：鈣質攝取[6] 、抽煙[7]、過量酒精攝取[8]、生理活動降低[9]。

骨骼是人體最具彈力且穩定的組織，也是生物體中第一個被觀察到有循環置換現象的組織，所以又是個動態組織(dynamic tissue)，在激素與物理因子的控制下，不斷地形成與毀壞，

以修改骨骼的架構使符合物理因素的壓迫[10,11]。骨骼並不是一種均勻的固態物質，在其堅硬的成份間都有一些空隙的存在，這些空隙是提供骨細胞養份的血管通道，若根據這些孔隙的大小及分佈的位置來看，骨骼可分為緻密的緻密骨(cortical bone)和內層較鬆散的樑狀骨(trabecular bone)兩種。

如(圖 2-5)，緻密骨見於長骨的骨幹和扁平骨的表層，由大量無機礦物質緊密地聚集形成，其孔隙較小且少，覆蓋於樑狀骨的外面，因此其特性是堅固、機械強度高、具有支持及保護作用。

如(圖 2-6)則為單一骨元各部份放大示意圖。相形之下，海綿骨的骨組織中孔隙較大且多，存在於大部份的短骨、扁平骨、不規則形狀骨的硬組織中，是由大量針狀或月狀的骨小樑(trabecula)相互連接而成的多孔網狀結構，網孔中充滿著骨髓，其中紅骨髓乃具有造血的作用[10]。骨小樑普遍順應最大應力和張力方向排列，而海綿骨內的膠原纖維與骨小樑的縱軸平行排列，許多膠原纖維穿過板間區，這種排列可增加骨骼對機械外力的抵抗。

在無機鹽類的組成物中，最主要是由未完全結晶的缺鈣氫氧基磷灰石 (calcium-deficient HAP)所構成[12];而有機基質則為網狀構造，主要包括大約 90%的膠原蛋白(collagen)、1%的葡萄糖胺聚合醣(glycosminoglycan)以及 5%左右的其他蛋白質，如(圖 2-7)。

發生骨質疏鬆症(osteoporosis)的骨頭，其樑狀骨會由原本較緻密的多孔結構變為疏鬆的網狀結構，而且質量也會減輕 [11,13]，因此當骨頭受外力時，很容易因為結構的支撐力不足而發生骨折。骨質疏鬆的現象在切除卵巢手術後的小鼠亦可觀察到，切卵巢後的小鼠由於雌性激素迅速下降，骨頭中的鈣磷大量流失而發生骨質疏鬆症，由高解析度微型 X 光電腦斷層掃描系統中 μCT)掃描可發現切除卵巢鼠骨之樑狀骨大量流失，但其緻密骨部份則無太大差異，如(圖 2-8) [14]。

骨質流失將影響骨骼強度，而利用骨礦物質密度 (bone mineral density, BMD) 的量測來評估骨質疏鬆是一個有效的方法。目前在臨床上有幾種常用的骨礦物質密度量測方法，包括單光子吸收測量 (single-photon absorptiometry)、雙能量 X 光吸收測量 (dual-energy X-ray absorptiometry) 及電腦斷層掃描 (computed tomographic, CT)。單光子吸收測量早在四十幾年前即開始使用，所用之能源通常為放射性同位素 (如 I-125)，利用其光子能量被軟組織及骨骼吸收之程度不同，藉以計算骨密度 [15]。雙能量 X 光吸收測量利用放射性 X 光測量骨礦物密度，因其光子流量強，對骨骼邊緣有較準確的測量，且還可預測骨折危險性。2003 年由 Rubin 學者以雙能量 X 光吸收譜儀掃描骨骼影像，並測量人類正常骨與骨質疏鬆骨之礦物質密度差異；研究發現正常骨之礦物密度值約為 0.580 mg/mm^3，而骨質疏鬆骨的礦物質密度僅約為 0.230 (女性) 至 0.273 mg/mm^3 (男性) [16]。Somerville 學者在 2004 年以高解析 X 光微型電腦斷層掃描 (Micro-CT) 研究不同年齡之 C57B1/6 鼠，分析其脛骨骨礦物質密度(BMD)，發現雄性鼠之骨礦物質密度較雌性鼠高，且成鼠又高於幼鼠 [17]。

2005 年 Hengsberger 等人做了一個有趣的實驗，他將卵巢切除(ovariectomy)及假手術(sham)兩種不同小鼠分別餵食 2.5%及 15%之同熱量酪蛋白(casein)，使得卵巢切除鼠骨頭之機械性質有更明顯低於假手術鼠之趨勢。而後將卵巢切除小鼠分成兩組，一組為對照組，另一組除了原本的 2.5%酪蛋白之外，增加了 5%必需胺基酸(essential amino acids)，比較其機械性質之差異性。發現卵巢切除之小鼠在脊椎骨三個不同部位機械性質皆比假手術鼠為低，顯示卵巢切除的確會造成骨質流失而使機械性質下降。餵食 5%必需胺基酸使機械性質些微上

升，其對於骨質疏鬆的治療是有幫助的[18]。

而中興大學實驗室用以治療骨質疏鬆小鼠的是酒精類的發酵乳，源起於北高加索山區，傳說具有醫療和保健的效用，在舊蘇聯時代，使用最為普遍，年產一百二十萬公噸以上。目前，在一些歐美國家和日本的市場上，可以買到相關產品[19]。**諾貝爾醫學獎得主－免疫學家梅契尼柯夫(Elias Metchnikoff) 1908 年發表「克弗爾之有效性、特性與長壽的關係」，從而引發克弗爾的研究風潮。克弗爾主要係由克弗爾菌元－克弗爾粒(kefir grain)發酵各類乳品而成，除具有一般發酵乳風味外，更含有微量酒精與二氧化碳所產生之獨特口感，具有乳香檳之稱。克弗爾菌元由乳酸菌和酵母菌共組共生菌相(symbiotic yeast-bacteria community)，因氣候、培養條件及乳原料不同，各區菌元組成有差異。主要菌群包括乳酸桿菌 *Lactobacillus*，如 *Lb. acidophilus*、*Lb. fermentum*、*Lb. casei*、*Lb. helveticus*、*Lb. plantarum*、*Lb. brevis*、 *Lb. kefir*、*Lb. casei* subsp. *rhamnosus* 等；白念球菌 *Leuconostoc*，如 *Leu. mesenteroides*；其他如 *Lacotococcus lactis* subsp. *cremoris*、*Streptococcus thermophilus* 等。酵母菌如 *Saccharomyces cerevisiae*、*Sac. pastorianus*、*Kluyveromyces lactis* var. *lactis*、*Klu. marxianus*、*Pichia fermentans* 、*Candida maris* 、*Can. kefyr* 等等。克弗爾經多方研究，已是公認由細菌與酵母菌共組的益生菌。克弗爾的保健機能性研究也已多被驗證，包括改善腸內菌相、改善乳糖不耐症、提升免疫機能、抑菌活性、抗腫瘤活性、抗氧化作用 、降膽固醇作用等。**

目前許多國家皆有許多克弗爾相關商品上市，包括益生菌粉製劑、發酵飲品、乾酪、麵包、甜點等，應用十分廣泛。眾所皆知，天然菌元－克弗爾粒之菌相因來源與培養條件而異，然其克弗爾食用安全性卻也被多數國家所認可。根據 FAO 與 WHO 共同組成的食品法典委員會（Codex Alimentarius Commission, CAC)所公布的食品質量標準，發酵乳製品類為 Codex Standard (Codex Stan 243-2003)。內容載明發酵乳(fermented milk)包括 Yoghurt、Alternate culture yoghurt、Acidophilus milk、Kefir、Kumys 等類別，如下所述。顯見 kefir grain 是發酵乳品所認可之發酵菌元。

Certain Fermented Milks are characterized by specific starter culture(s) used for fermentation as follows:

**Yoghurt:**	**Symbiotic cultures of *Streptococcus thermophilus* and *Lactobacillus delbrueckii* subsp. *bulgaricus.***
**Alternate Culture Yoghurt:**	**Cultures of *Streptococcus thermophilus* and any *Lactobacillus* species.**
**Acidophilus Milk:**	***Lactobacillus acidophilus.***
**Kefir:**	**Starter culture prepared from kefir grains, *Lactobacillus kefiri*, species of the genera *Leuconostoc*, *Lactococcus* and *Acetobacter* growing in a strong specific relationship. Kefir grains constitute both lactose fermenting yeasts (*Kluyveromyces marxianus*) and non- lactose-fermenting yeasts (*Saccharomyces unisporus, Saccharomyces cerevisiae* and *Saccharomyces exiguus*).**
**Kumys:**	***Lactobacillus delbrueckii* subsp. *bulgaricus* and *Kluyveromyces marxianus.***

Other microorganisms than those constituting the specific starter culture(s) specified above may be added. (Codex Stan 243-2003)

本計畫所使用之菌元源於台灣大學動物科學系　林慶文教授乳品加工實驗室。計畫總主持人自碩士班開始即跟隨林教授從事益生菌與乳成分機能性研究、發酵乳品開發等研究工作，迄今歷經十六年，對 **kefir grain** 之應用與特性已累積相當之研究經驗與成果。同時，林教授實驗室也是國內發表 **kefir** 相關國內外文獻最多之學者。有關菌種組成研究，曾自此菌元分離純化乳酸菌共計 **7** 株，包括有乳酸桿菌屬 **1** 株及白念球菌屬 **6** 株。經進一步鑑定後，確定為 ***Lactobacillus helveticus*** **1** 株與 ***Leuconostoc mesenteroides*** **6** 株。***Leuconostoc mesenteroides*** 在發酵乳中扮演重要的角色，維生成聯乙醯等香氣物質之主要微生物。且由於它們的抗菌作用及不易受噬菌體攻擊等特性，因此在發酵菌元中佔有重要地位。酵母菌在克弗爾中也扮演極為重要角色，因其代謝產生的乙醇及二氧化碳，使克弗爾具有特殊的口感與風味。此菌元共計分離純化出 **8** 株酵母菌，鑑定結果其中 **6** 株酵母菌為 ***Kluyveromyces marxianus***，**2** 株為 ***Pichia fermentans***。並驗證克弗爾具有抗菌 **(antimicrobial)**、抗突變 **(antimutagenic)**、抗氧化 **(antioxidant)** 及降膽固醇 **(hypocholesterolaemic)**。此菌相與功能性研究成果已被最新的 **kefir review** 期刊所引用。

綜上所述，本研究先期成果及本計畫擬使用之 **kefir grain**，無論菌相或功能性皆已具備

良好的研究基礎，相關成果也為國際 kefir 研究學者所認同引用。有研究發現以 *Lactobacillus helveticus* 這株菌的發酵乳會使停經婦女鈣質的正向代謝，也就是鈣質流失趨緩。另一項研究證實發酵乳對於停經婦女會降低其夜間骨質吸收。

本實驗室近期就對於卵巢切除骨質疏鬆鼠的緻密骨組織微結構與機械性質之研究，發現圖 2-9 為在不同條件老鼠股骨之骨幹緻密骨 Micro-CT 3-D 影像圖，基本上各條件鼠之緻密骨形貌相近，並無明顯之差異。但是由圖 2-10 所示之樑狀骨 3-D 影像圖中可發現，正常鼠之樑狀骨結構緊密、單一樑狀骨較粗；而假手術鼠樑狀骨結構與正常鼠類似，但較正常鼠些微鬆散，且單一樑狀骨亦較正常鼠略細。切除卵巢鼠之樑狀骨結構則明顯較正常鼠鬆散，且其單一樑狀骨較正常鼠細許多，顯示 切除卵巢鼠因切除卵巢後受體內雌激素下降之影響，使成骨細胞 (osteoblast) 與破骨細胞 (osteoclast) 作用失衡，導致骨骼吸收速率大於形成速率，進而造成骨質流失。雌激素有抵抗甲狀旁腺激素排鈣的作用，當雌激素下降時，此抵抗能力將減弱，於是骨骼中之鈣質將會被分解、溶解入血液中，並藉由尿液排出 [20]。而發酵乳鼠之樑狀骨結構與正常鼠相當類似，表示切除卵巢後的老鼠以發酵乳營養劑餵養，其骨骼可獲得相當程度的養分補充，並可有效防止骨質流失，使其樑狀骨結構可維持與正常鼠一樣。各種不同條件鼠股骨利用高解析 X 光微型電腦斷層掃瞄 (Micro-CT) 所得之影像，經重組後以電腦軟體 (CTAn) 計算其骨礦物質密度 (與標準試片比較值)，如圖 2-11 所示。發酵乳鼠的骨礦物質密度約為 560 mg/cm^3，亦保持與正常鼠及假手術鼠相同的數值，表示發酵乳營養補充對抑制切除卵巢鼠之骨質流失有明顯的成效，可充分補充流失之鈣質。

而其緻密骨奈米機械性質則以奈米壓痕測試儀分析各種不同條件鼠股骨緻密骨內至外側機械性質之變化。顯示切除卵巢後以發酵乳餵養的老鼠，其骨質流失可受到控制或可補足其流失之骨質。相較於正常鼠及假手術鼠，發酵乳鼠緻密骨內側之彈性模數與硬度甚至較外側為高，以此結果推論，發酵乳可有效幫助骨頭吸收鈣和磷等養分，加速內側骨基質之礦化，進而強化骨頭的機械強度。圖 2-12 至 2-13 為各種不同條件鼠股骨緻密骨內側至外側之平均彈性模數與硬度值及機械性質，由此圖可以很清楚看出上述機械性質隨處理條件之變化。由上述實驗證明發酵乳有效促進切除卵巢鼠對鈣磷的吸收與補充，可抑制其骨質流失，使其骨礦物質密度及機械性質皆可維持與正常鼠相當。

(A) (B)

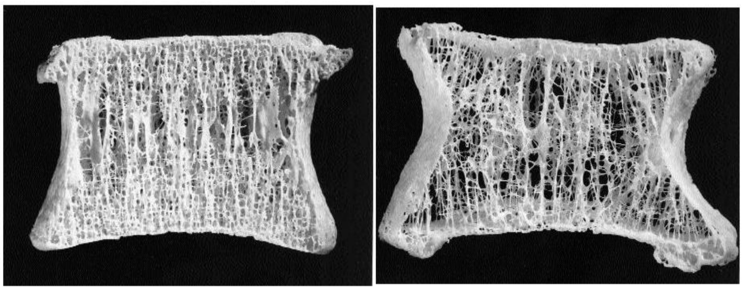

圖 2-1、正常骨質與疏鬆骨質之比較(Mosekilde ,1998)。(A)正常骨質：正常的人體骨骼切面，骨樑非常緊密，結構也相當完整。(B)疏鬆骨質：骨質疏鬆症患者的骨骼切面，骨樑顯得相當疏鬆，而結構也呈現斷裂和破碎的情形。

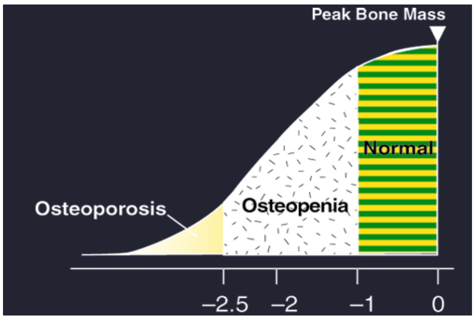

圖 2-2、世界衛生組織對骨質疏鬆症分級方式(WHO, 1994)。Normal：骨質密度高於年輕健康成人平均值-1 個標準差以上。osteopenia：骨質密度介於年輕健康成人平均值-1 至-2.5 個標準差之間。osteoporosis：骨質密度低於年輕健康成人平均值-2.5 個標準差以下。

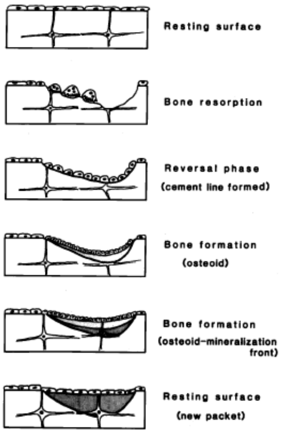

圖2-3、骨質的再塑過程(Mosekilde L.,1998)。活化期：osteoclast活化，在休止期表面osteoblast以扁平狀排列，接受刺激後，變成立方體出現空隙，來自骨髓的幹細胞proosteoclast分化，遊走到表面；吸收期：osteoclast活化，持續約10天吸收期，開始在骨表面溶解、消化、吸收；逆轉期：當osteoclast吸收至一定量停止，股吸收表面形成一道線，是骨吸收與骨形成過渡期；形成期：osteoblast在骨吸收處開始製造新骨質；靜止期：新生骨補足骨吸收部份，骨表面形成休止狀態。

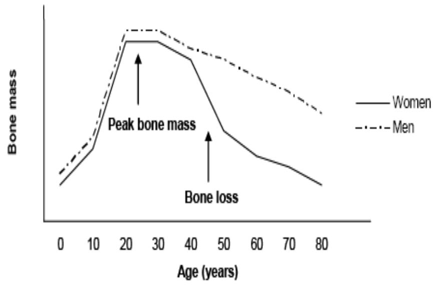

圖 2-4、男性與女性骨密度達高峰與骨質流失之比較(Teegarden *et al.*,1995)。

骨質量在幼年，青春期逐漸增加，骨骼隨大小和密度增加，約 90%的骨質量高峰在男生 20 歲女生 18 歲達高峰，在 30 歲達最大強度與密度，為骨密度高鋒。在 30 歲女性與停經期，骨密度有逐漸下降趨勢，停經期初期，骨質流失快速，之後數年緩慢持續流失。

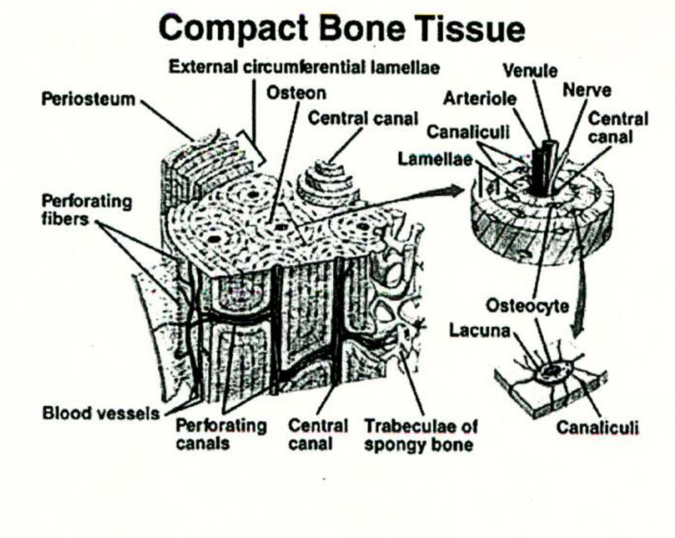

圖 2-1 長骨骨幹 (diaphysis) 壁的模式圖 [5]

圖 2-5

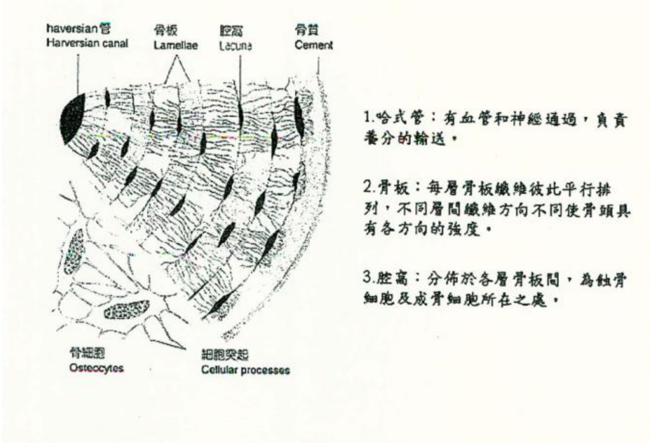

圖 2-2 板狀骨結構簡介 [5]

圖 2-6

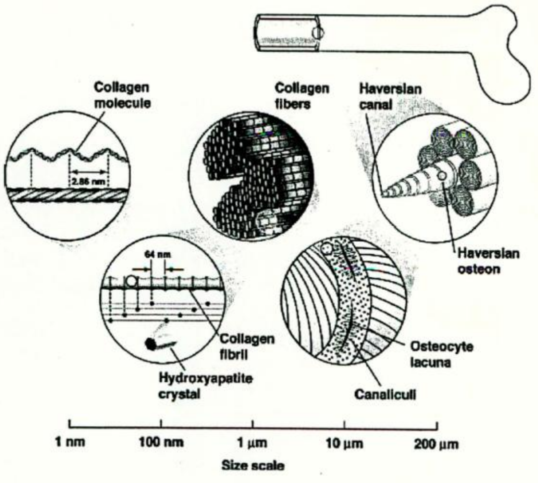

圖 2-3 緻密骨不同尺度下的多層次結構 [8]

圖 2-7

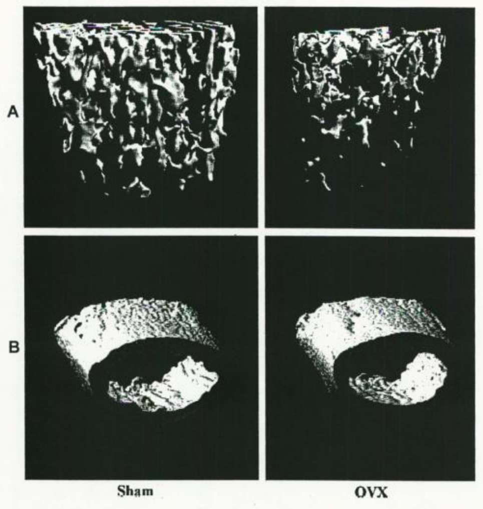

圖 2-15 利用微型 X 光電腦斷層掃瞄分析卵巢切除小鼠之骨質變化。A：樑狀骨經 μCT 掃描之影像 B：緻密骨經 μCT 掃描之影像；Sham： 未切除卵巢之對照組小鼠；OVX： 切除卵巢之實驗組小鼠 [23]

圖 2-8

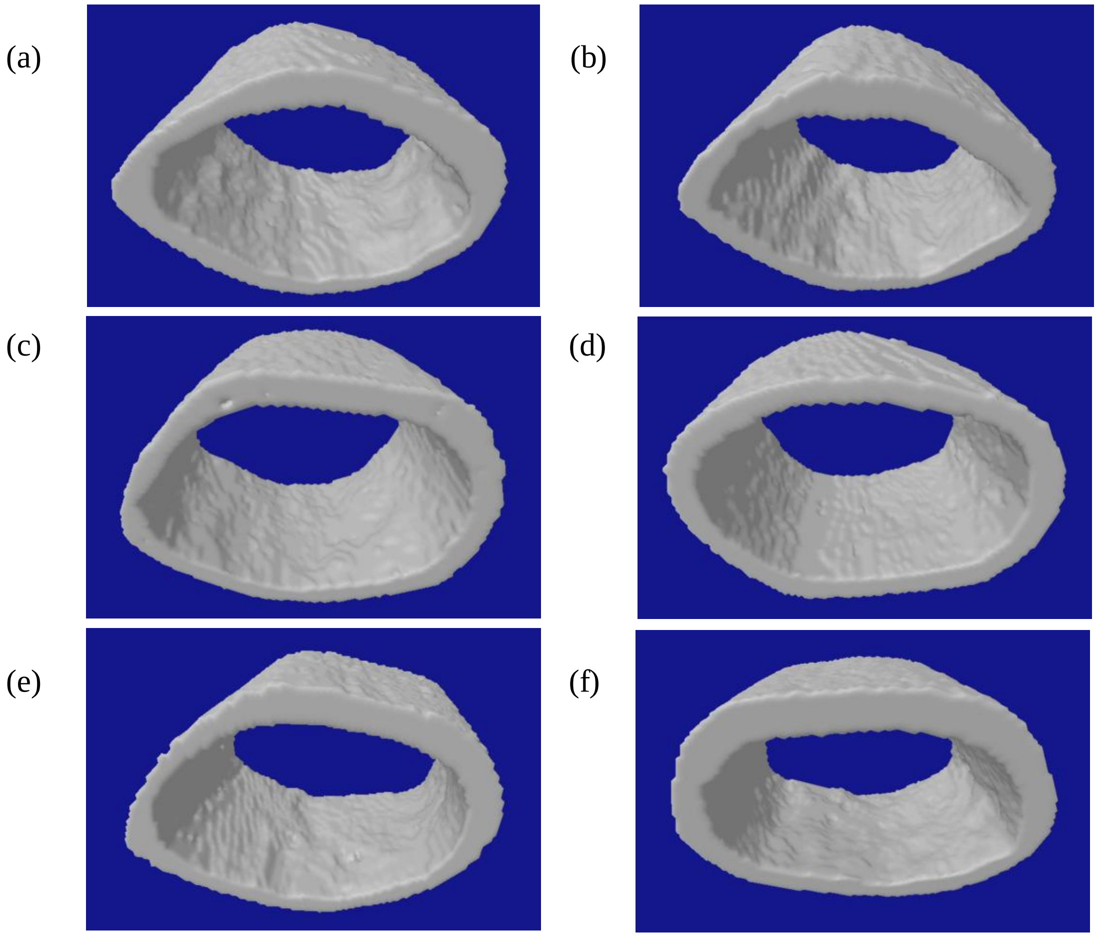

圖 2-9 各條件鼠股骨之骨幹緻密骨 Micro-CT 3-D 影像圖：(a) 正常鼠、(b) 假手術鼠、(c) 切除卵巢鼠、(d) A 群酪蛋白鼠、(e) B 群酪蛋白鼠、(f) 發酵乳鼠。

(a) (b) (c) (d) (e) (f)

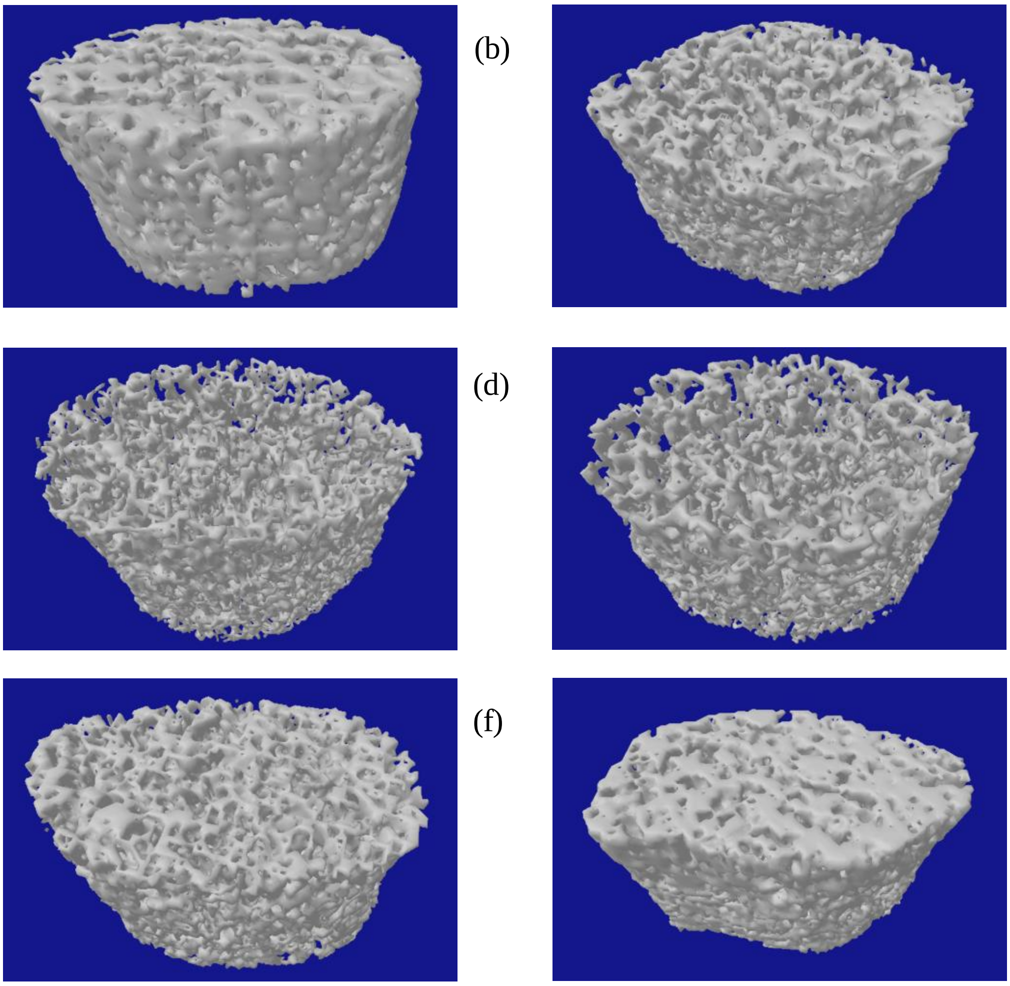

圖 2-10 各條件鼠股骨之骨幹樑狀骨 Micro-CT 3-D 影像圖：(a) 正常鼠、(b) 假手術鼠、(c) 切除卵巢鼠、(d) A 群酪蛋白鼠、(e) B 群酪蛋白鼠、(f) 發酵乳鼠。

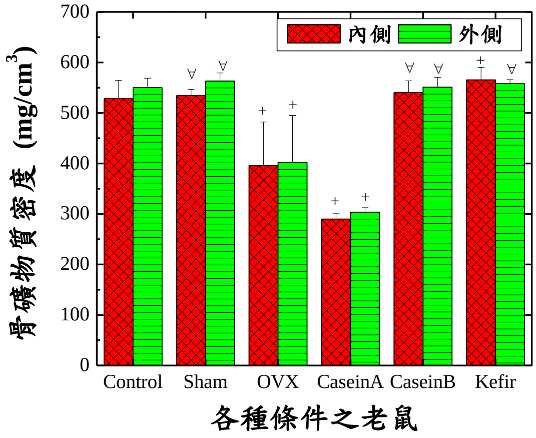

圖 2-11 不同條件鼠之股骨緻密骨內外側礦物質密度 (CTL：正常鼠，SHM：假手術鼠，OVX：切除卵巢鼠，CSN：酪蛋白鼠，YMK：發酵乳鼠) (*為 $p < 0.05$, +為 $p < 0.01$, ∀為 $p > 0.05$)。

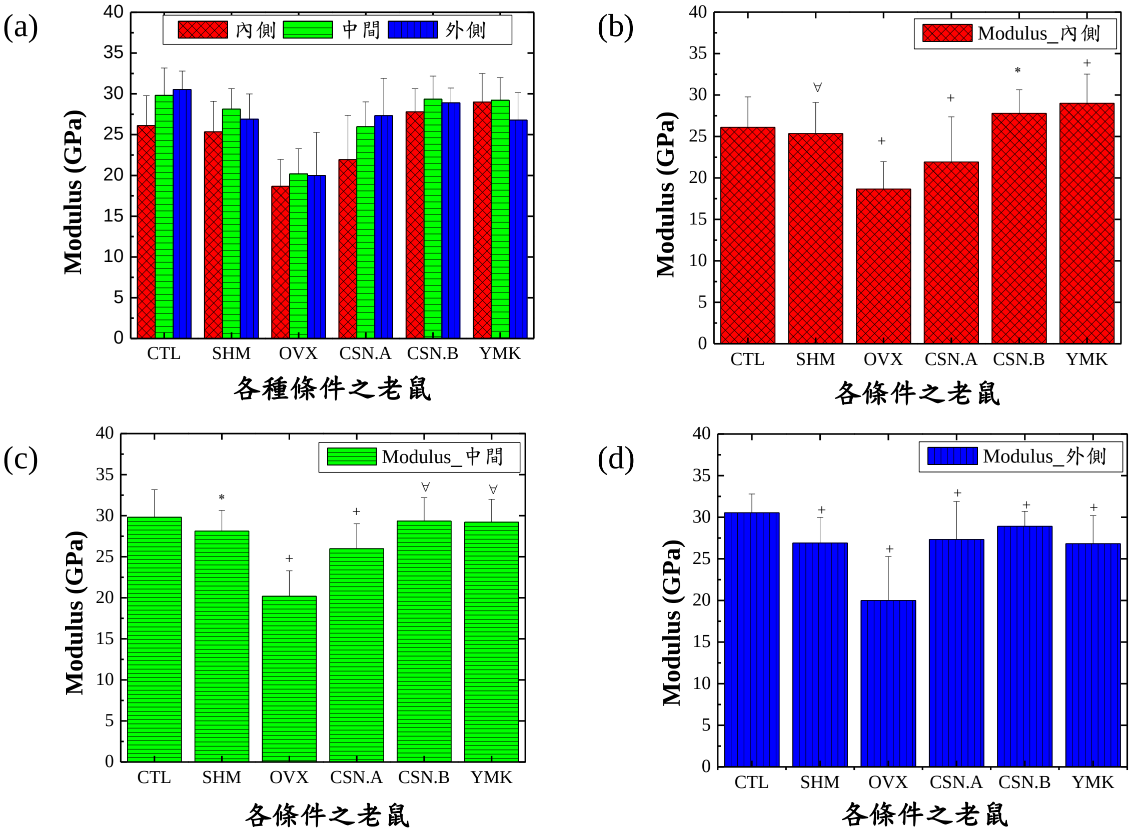

圖 2-12 不同條件鼠股骨緻密骨內側至外側之機械性質：(a) 內中外側彈性模數、(b) 內側彈性模數、(c) 中間彈性模數、(d) 外側彈性模數 (CTL：正常鼠，SHM：假手術鼠，OVX：切除卵巢鼠，CSN：酪蛋白鼠，YMK：發酵乳鼠) (*為 $p < 0.05$, +為 $p < 0.01$, ∀為 $p > 0.05$)。

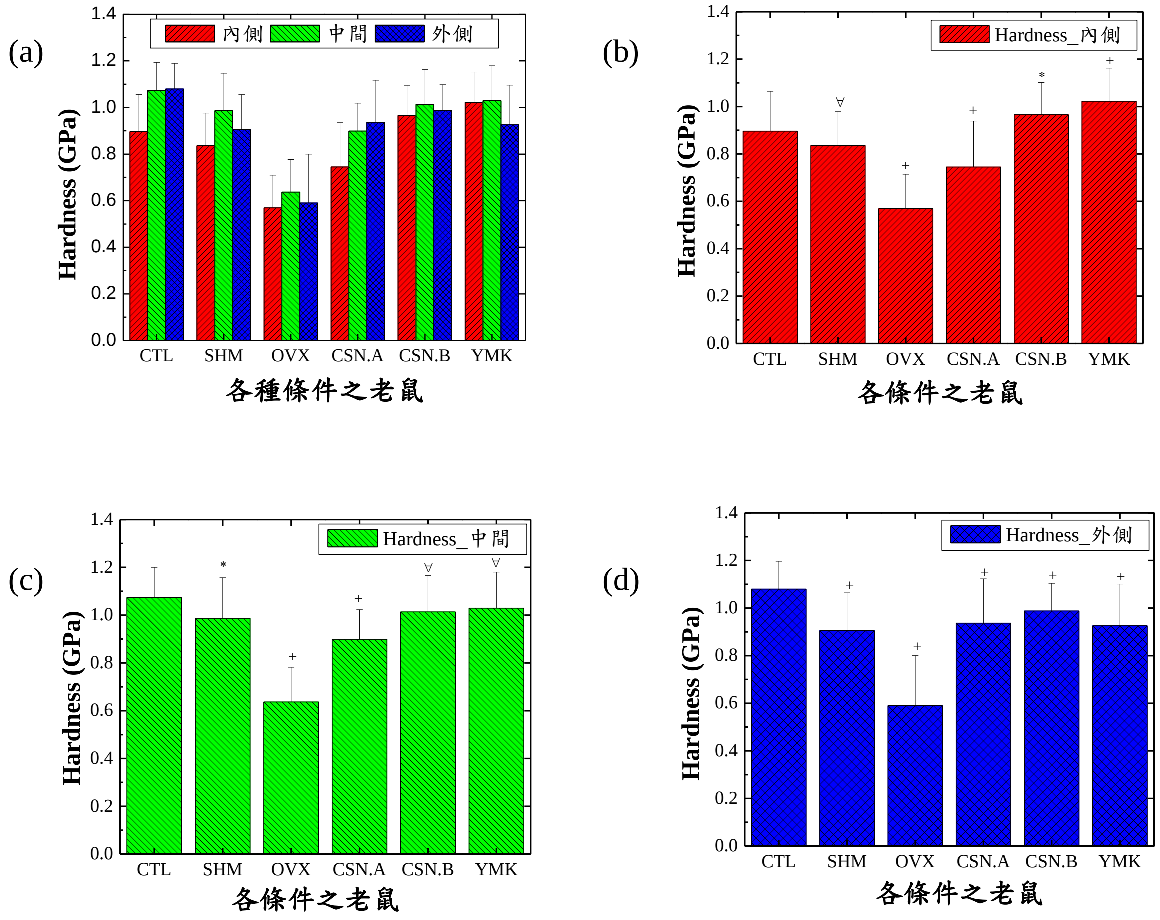

圖 2-13 不同條件鼠股骨緻密骨內側至外側之機械性質：(a) 內中外側硬度、(b) 內側硬度、(c) 中間硬度、(d) 外側硬度 (CTL：正常鼠，SHM：假手術鼠，OVX：切除卵巢鼠，CSN：酪蛋白鼠，YMK：發酵乳鼠) (*為 $p < 0.05$, +為 $p < 0.01$, ∀為 $p > 0.05$)。

二、計畫目的：請分項具體描述本計畫預定達成之目標以及計畫完成之工作項目，應避免敘述內容流於空泛、形式；若屬跨年度之中、長期跨年計畫，應再列述全程計畫之總目標及分年計劃之目的。

骨頭中鈣和磷的流失，將發生所謂的骨質疏鬆症 (osteoporosis)，骨質流失後的骨頭受外力衝擊時較一般正常骨頭容易脆裂，而其中是否經由飲食或藥物的使用，而抑制鈣和磷的流失，甚至增加骨質密度是許多人所關切的課題。基於中興大學實驗室用以治療骨質疏鬆小鼠的是酒精類的發酵乳經驗，對於改善骨質疏鬆的效果良好。

因此，本實驗的目的在於比較食用發酵乳與未食用發酵乳之患者，在治療三個月與六個月之後，以雙能量式 X 光骨質密度檢查 (DEXA)評估病人治療後病患骨質密度以及生化檢驗；如血中之鈣磷濃度、副甲狀腺素(PTH)、Osteocalcin、Deoxy-pyridnoline cross-links 改善程度。

三、實行方法暨進行步驟：請詳列實施本年度計畫所採用之方法及步驟；研究計劃應予詳加說明研究設計、資料收集以及分析方法。

試驗設計

(一)受試者

1.納入標準: 骨質疏鬆及其造成相關併發症的病人。

2.排除標準: (1)雙側卵巢切除(bilateral ovariectomies)者、40 歲以前自然停經者、(2)服用藥物或服用骨頭代謝之藥物，例如：糖皮質類固醇(glucocorticoid)、甲狀腺素(thyroxin)、抗癲癇劑(antiepileptics)、二磷酸酐類藥物(bisphosphonates)、抑鈣素(calcitonin)、荷爾蒙治療(hormone replacement therapy)超過 4 個月以上，(3)體重超過 100 公斤，(4)有以下疾病史者：原發性副甲狀腺機能亢進症(primary Hyperparathyroidism)、甲狀腺機能亢進症(hyperthyroidism)、糖尿病(diabetes)、肝硬化(cirrhosis)、腎衰竭(kidney failure)，這些排除的族群皆有醫療病史，或實驗室報告確立者。

(二)研究用藥及給藥方式

被診斷為骨質疏鬆症病患分為兩組：

1. 發酵乳治療組

病患分為兩種劑量 每天須服用 1600mg 及 2400mg 之發酵乳，並補充 1500mg 之鈣質及補充 400 至 800 單位的維生素Ｄ

2.CONTROL 組：病患只補充 1500mg 之鈣質及補充 400 至 800 單位的維生素Ｄ。

(三)臨床評估

1.以雙能量式 X 光骨質密度檢查 (DEXA)評估病人治療後三個月及半年後病患骨質密度改善程度。

2.生化檢驗:如血中之鈣磷濃度、副甲狀腺素(PTH)、Osteocalcin、Deoxy-pyridnoline cross-links。

(四)統計分析

兩組病人的年齡、性別、身高、體重、骨質疏鬆嚴重度用 student’s *t*-tests 來比較是否有差異性，並用 ANOVA 來比較兩組骨質密度，P＜0.05 認為有統計上之意義。

四、重要參考文獻：請依一般科學論文之參考文獻撰寫範例，逐一列出本計畫所引用之參考文獻，並於計畫內容引用處加註標記之。

[1] Cummings, SR,and Melton LJ.2002.Epidemiology and outcomes of osteoporosis fractures.Lancet 359:1761-1767.

[2] Cummings SR, Black DM, Nevitt MC, Browner W, Cauley J,Ensrud K,Genant HK, Palermo L, Scott J,and Vogt TM.1993. Bone density at various sites for prediction of hip fractures. The study of osteoporotic fractures research group. Lancet 341:72-75.

[3] Dargent-Molina P, Favier F, Grandjean H, Baudoin C, Schott AM,Hausherr E, Meunier PJ,and Breart G.1996. Fall-related factors and risk of hip fracture: The EPIDOS prospective study.Lancet 348:145-149.

[4] Faulkner KG, Cummings SR, Black D, Palermo L, Gluer CC,and Genant HK. 1993.Simple measurement of femoral geometry predicts hip fracture: The study of osteoporotic fractures. J. Bone. Miner. Res. 8:1211-1217.

[5] Yilmaz D, Ersoy B, Bilgin E, Gumuser G, Onur E and Pinar ED.2005.Bone mineral density in girls and boys at different pubertal stages: relation with gonadal steroids, bone formation markers, and growth parameters. J.Bone Miner. Metab. 23:476-82.

[6] Holbrook TL, Barrett-Connor E, and Wingard DL.1988. Dietary calcium and risk of hip fracture:14 year prospective population study.Lancet.2：1046-1049.

[7] Law MR, and Hackshaw AK. 1997. A meta-analysis of cigarette smoking, bone mineral density and risk of hip fracture: Recognition of a major effect. Br.Med.J. 315:841-846.

[8] Feitelberg S, Epstein S, Ismail F,and D'Amanda C. 1987. Deranged bone mineral metabolism in chronic alcoholism. Metabolism 36:322-326.

[9] Cooper C, Barker DJP,and Wickham C. 1988. Physical activity, muscle strength, and calcium intake in fracture of the proximal femur in britain.Br. Med. J. 297:1443-1446.

[10] L. C. Junqueira, J. Carneiro, and R. 0. Kelley, "Basic Histology ninth edition"，合記圖書出版社, (2000).

[11] Stavros C. Manolagas, and R. L. Jilka, "Bone Marrow, Cytokines, and Bone Remodeling", *N. Engl. J. Med.,* Vol. 332, No. 5, p. 305-311 (2006).

[12] Turek SL. "Orthopaedics Principles and Their Applications", J.B. Lippincott Company, Vol. 1, p. 31-100 (1984).

[13] P. Zioupos and J. D. Currey, "Changes in the Stiffness, Strength, and Toughness of Human Cortical Bone With Age", *Bone,* Vol. 22, No. 1, p. 57-66 (1998).

[14] A. Xiang, M. Kanematsu, M. Mitamura, H. Kikkawa, S. Asano, and M. Kinoshita, "Analysis of Change Patterns of Microcomputed Tomography 3-Dimensional Bone Parameters as a High-Throughput Tool to Evaluate Antiosteoporotic Effects of Agents at an Early Stage of Ovariectomy-Induced Osteoporosis in Mice", *Invest. Radiol.,* Vol. 41, No. 9, p. 704-712 (2006).

[15] B.L. Riggs, and L.J. Melton, “Osteoporosis: etiology, diagnosis, and management.”, Raven Press, New York, (1988). 楊榮森 編譯，“骨質疏鬆症:病因．診斷．治療”，合計圖書出版社，台北市 (1997) p. 1-73, 261-267, 369-375.

[16] M.A. Rubin, I. Jasiuk, J. Taylor, J. Rubin, “TEM analysis of the nanostructure of normal and osteoporotic human trabecular bone”, *Bone*, 33 (2003) 270-282.

[17] J.M. Somerville, R.M. Aspden, K.E. Armour, K.J. Armour, and D.M. Reid, “Growth of C57Bl/6 Mice and the Material and Mechanical Properties of Cortical Bone from the Tibia”, *Calcif. Tissue Int.*, 74 (2004) 469–475.

[18] S. Hengsberger, P. Ammann, B. Legros, R. Rizzoli, and P. Zysset, "Intrinsic bone tissue properties in adult rat vertebrae:modulation by dietary protein", *Bone*, Vol. 36, p. 134-141 (2005).

[19] 郭卿雲，"畜產食品科技 另類的發酵乳 克弗爾"科學發展.

[20] P. Braidotti, E. Bemporad, T.D. Alessio, S.A. Sciuto, and T. Stagni “Tensile experiments and SEM fractography on bovine subchondral bone” *J. Biomech.*, 33 (2000) 1153-1157.

五、預計成果概要：請列述預定於執行期限內完成之工作項目及具體成果；若屬中、長程跨年度之研究計劃，請分年度列述。
一、預期完成項目：<br>1. 取得發酵乳治療三及六個月後骨密度的數值。<br>2. 取得病患接受治療前後，其有關骨質流失相關之生化指標的數據。<br><br>二、預期成果：<br>1. 經由發酵乳治療的病患，其骨密度(DEXA)應有改善，其生化檢驗亦可能骨質流失趨緩。<br>2. 無發酵乳治療組，骨質沒有改善甚至有流失現象。
